# Giant Circularly Polarized Luminescence Driven by Excited‐State Hybridization Between Molecular Emitters and Chiral Environments

**DOI:** 10.1002/adma.202506941

**Published:** 2025-07-16

**Authors:** Li Wan, Eunkyung Cho, Rui Zhang, Theis Brock‐Nannestad, Zhaohui Wang, Jean‐Luc Brédas, Veaceslav Coropceanu, Feng Gao

**Affiliations:** ^1^ Department of Physics Chemistry and Biology (IFM) Linköping University Linköping 58432 Sweden; ^2^ Max Planck Institute of Microstructure Physics 06120 Halle Germany; ^3^ Divison of Energy and Environmental Technology DGIST Daegu 42988 Republic of Korea; ^4^ Department of Chemistry and Biochemistry The University of Arizona Tucson AZ 85721‐0041 USA; ^5^ Department of Chemistry University of Copenhagen Copenhagen 2100 Denmark; ^6^ Key Laboratory of Organic Optoelectronics and Molecular Engineering Department of Chemistry Tsinghua University Beijing 100084 China

**Keywords:** chirality amplification, chirality, circularly polarized luminescence, excited state hybridization

## Abstract

Circularly polarized (CP) light is extensively used in various fields such as asymmetrical synthesis, sensing, and advanced displays. Consequently, significant efforts have been made to develop chiral materials that intrinsically emit CP light with a large dissymmetry factor (*g*‐factor). It is widely considered that the dissymmetry factor for individual organic emitters, due to the amplitude offset between their electric and magnetic transition dipole moments, is limited to ≈10^−2^, which is inadequate for practical applications. Recent efforts to enhance CP light emission have therefore focused on amplifying the dissymmetry of circularly polarized luminescence (CPL), often via specific energy transfer processes. Here, a fundamental mechanism is discovered – excited‐state hybridization, which amplifies CPL through excitonic coupling without relying on energy transfer processes. Through this wavefunction hybridization, both the amplitude and sign of the rotatory strength related to the molecular emitter's electronic transition are modified to align with its chiral environment, remarkably boosting the CP luminescence from an intrinsic dissymmetry factor of −10^−3^ up to +0.40. This breakthrough allows for more versatile design strategies for chiral emissive systems, moving beyond designs limited to energy transfer processes and paving the way for new approaches to achieve strong CP emissive materials.

## Graphical abstract

A fundamental chiroptical response amplification mechanism is revealed to massively enhance molecular helicene chiral emitters. The luminescent dissymmetry factor of the helicene emitter is amplified by 133 times through excited‐state hybridization with a polymeric chiral environment. The amplification mechanism does not rely on a specific energy transfer process, therefore offering new paths for the design of chiral emissive systems.

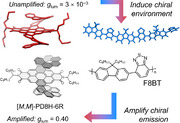

## 1 Introduction

Chirality characterizes a fundamental asymmetry of molecules, where a pair of the existing forms of structures (i.e., enantiomers) cannot be superposed on each other. Chiral systems exhibit additional photophysical information when coupled with circularly polarized light, including selectively chiral light absorption [i.e., circular dichroism (CD)^[^ ^1^ ^]^] and circularly polarized luminescence (CPL). However, generating strongly circularly polarized luminescence using intrinsically chiral materials is a challenging task.^[^ ^2^, ^3^, ^4^ ^]^ Addressing this challenge is definitely a key technical requirement for the commercialization of chiral light sources, particularly for their application in efficient display technologies.^[^ ^5^, ^6^, ^7^ ^]^

Though the chiroptical response can be *extrinsically* amplified with an external magnetic field^[^ ^8^ ^]^ and optical filter or equivalent liquid crystal layers,^[^ ^9^ ^]^ it is still extremely difficult to massively enhance the *intrinsic* chiral emission of a chiral emitter, which is associated directly with the material's electronic transitions.^[^ ^10^, ^11^, ^12^ ^]^ The parameter that is widely used in the community to quantify the polarization of a photophysical process is the dissymmetry factor (*g*‐factor).^[^ ^13^ ^]^ The molecular *g*‐factor for an electronic transition is proportional to the rotatory strength (*R*) of a transition as described below:^[^ ^14^ ^]^

(1) $$g=\frac{4R}{D}=\frac{4|\mu \left|\right|m|\cos\theta }{|\mu {|}^{2}+|m{|}^{2}}\;$$

where *D* is the dipole strength; ***µ*** and ***m*** represent the electric and magnetic transition dipole moments, respectively, with *θ* being the angle between them. For chiral emission, the experimental luminescence *g*‐factor (*g*_lum_) can be extracted from circularly polarized luminescence (CPL) spectra using the following equations:

(2) $$g_{\mathrm{lum}}=2\times \frac{I_{L}-I_{R}}{I_{L}+I_{R}}$$

where *I*_L_ and *I*_R_ represent the intensity fractions of the left‐ and right‐handed CPL, respectively. A fully circularly polarized emission thus leads to |*g*| = 2. For organic emitters, the amplitude of |μ| is generally ≈1000 times larger than that of | ***m*** |.^[^ ^14^ ^]^ Even when introducing extremely twisted structures in molecular backbones, the rotatory strength achieved in these emitters is still overwhelmed by *D*,^[^ ^15^ ^]^ resulting in *g*_lum_ values of state‐of‐the‐art chiral emissive organics of ≈10^−2^.^[^ ^4^, ^10^, ^11^, ^16^, ^17^ ^]^

In recent studies on amplified circularly polarized emission from organic molecules, a variety of mechanisms have been proposed, which were related to specific energy transfer processes, such as Förster Resonance Energy Transfer (FRET)^[^ ^18^ ^]^ and Dexter energy transfer driven Triplet‐Triplet Annihilation Upconversion (TTAU)^[^ ^19^, ^20^ ^]^ (see detailed comparisons in **Table** **1**). For example, the Fuchter group reported an amplified CPL from a superhelicene (oxa[7]superhelicene)^[^ ^18^ ^]^ by overlapping its absorption profile with the emission of a chiral polymer sensitizer/host^[^ ^18^ ^]^ and promoting the FRET process. In another case reported by the Liu and Duan group,^[^ ^19^, ^20^ ^]^ a binaphthyldiamine emitter was demonstrated to display an enhanced *g*_lum_ due to upconverted photoluminescence, assisted by Dexter energy transfer from an achiral Pt(II) sensitizer. While energy transfer is a key process in efficient light‐emitting systems across various optoelectronic applications,^[^ ^21^, ^22^, ^23^ ^]^ these observations could imply that the CPL amplification must be correlated with such mechanisms, thereby limiting the scope of novel chiral emitter‐host designs.

**Table 1:** Comparison of the electronic transitions in [*M*,*M*]‐PD8H‐6R without and with a chiral F8BT environment.

Molecular orientation	Dominant electronic transition	E [eV]	E [nm]	*f* ^a)^	|*μ*| [10^−20^ esu cm]	|*m*| [10^−20^ erg G^−1^]	cos [*θ* ^b)^]	*R* [10^−40^ erg esu cm G^−1^]	*g*
w/o F8BT	S_0_ → S_1_	2.38	521	0.127	375	0.99	−0.31	−114	−0.003
	S_0_ → S_2_	2.40	518	0.034	194	0.23	−1.00	−45	−0.005
	S_0_ → S_3_	2.41	513	0.004	63	0.64	−0.67	−27	−0.027
w/ F8BT	S_0_ → S_1_	2.36	526	0.001	36	2.77	0.80	81	0.243
	S_0_ → S_2_	2.36	525	0.055	247	3.41	0.53	450	0.029
	S_0_ → S_3_	2.38	521	0.156	416	3.02	0.07	86	0.002

^a)^ Oscillator strength of the electronic transition;

^b)^ Angle between ***μ*** and ***m***.

Here, we use an emissive double ^[^ ^8^ ^]^ helicene^[^ ^24^ ^]^ to demonstrate a CPL amplification in this molecular emitter from an intrinsic *g*_lum_ value of ≈−10^−3^ to an amplified *g*_lum_ value of ≈+0.40, which is induced by a chiral polymeric environment. The amplified CPL has a sign opposite to that of the unamplified CPL signals from the helicene emitters, pointing out that the photophysical origin of the amplification is not just a boost, but is associated with changes in the emitter's electronic structure. Further calculations indicate that the electronic structure of a chiral molecule changes substantially in a chiral environment compared to its isolated form, and the corresponding transition dissymmetry factors flip even without inverting the geometry of the chiral enantiomers. These investigations allow us to attribute the origin of the CPL amplification and sign inversion to an excited‐state hybridization mechanism rather than an energy transfer process. This new mechanism can greatly relax the material design requirements associated with FRET or TTAU processes and stimulate the exploration of novel strategies for designing strongly circularly polarized emissive organics.

## 2 Results and Discussion

**Figure 1:**
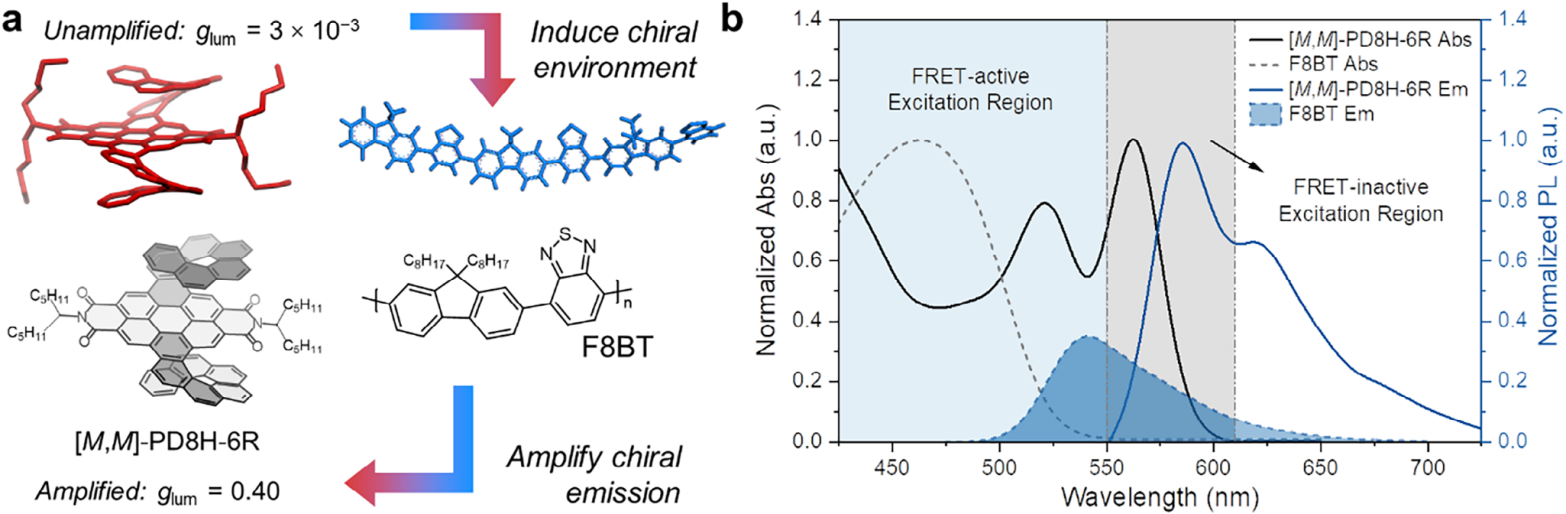
Schematic summary of the CPL amplification process. a) Molecular structures of the emitter [*M*,*M*]‐PD8H‐6R and the host matrix F8BT. b) Absorption and photoluminescence profiles of F8BT and [*M*,*M*]‐PD8H‐6R thin films. Regions are marked in blue and gray to highlight FRET activity when exciting a [*M*,*M*]‐PD8H‐6R:F8BT blend film at different wavelengths.

A double helicene (PD8H‐6R,^[^ ^24^ ^]^ **Figure** **1**) with a wide absorption window was selected for our spectroscopic investigations. This helicene features a structure consisting of two fused ^[^ ^8^ ^]^ helicene on each side of a perylene diimide (PDI) core, forming enantiomers [*M*,*M*]‐PD8H‐6R (Figure 1a) and [*P*,*P*]‐PD8H‐6R. These helicene moieties grant intrinsic molecular chirality to the PDI backbone; the lowest excited state corresponds to a helicene to PDI charge‐transfer‐like transition (Figures S1 and S2, Tables S2 and S3, Supporting Information), with an absorption profile peaking at λ = 562 nm in thin films and a strong photoluminescence (PL) signal at λ = 585 nm (Figure 1b). [*M*,*M*]‐PD8H‐6R^[^ ^24^ ^]^ demonstrates a photoluminescence quantum efficiency (PLQY) of ≈33% and a high *g*_abs_ of −1.2 × 10^−2^ in solution. After identifying PD8H‐6R as a promising emitter, we next focused on creating a chiral environment and selected a polyfluorene copolymer – poly(9,9‐dioctylfluorene‐alt‐benzothiadiazole) (F8BT, Figure 1a) as host material. There were two reasons for this choice: 1) Polyfluorenes including F8BT can be endowed with a strong chiroptical response when they are blended with chiral molecules;^[^ ^25^, ^26^, ^27^ ^]^ 2) F8BT has an absorption edge at ≈550 nm, thus leading to a large ≈60 nm FRET‐inactive spectral window for our photophysical investigations (Figure 1b).

**Figure 2:**
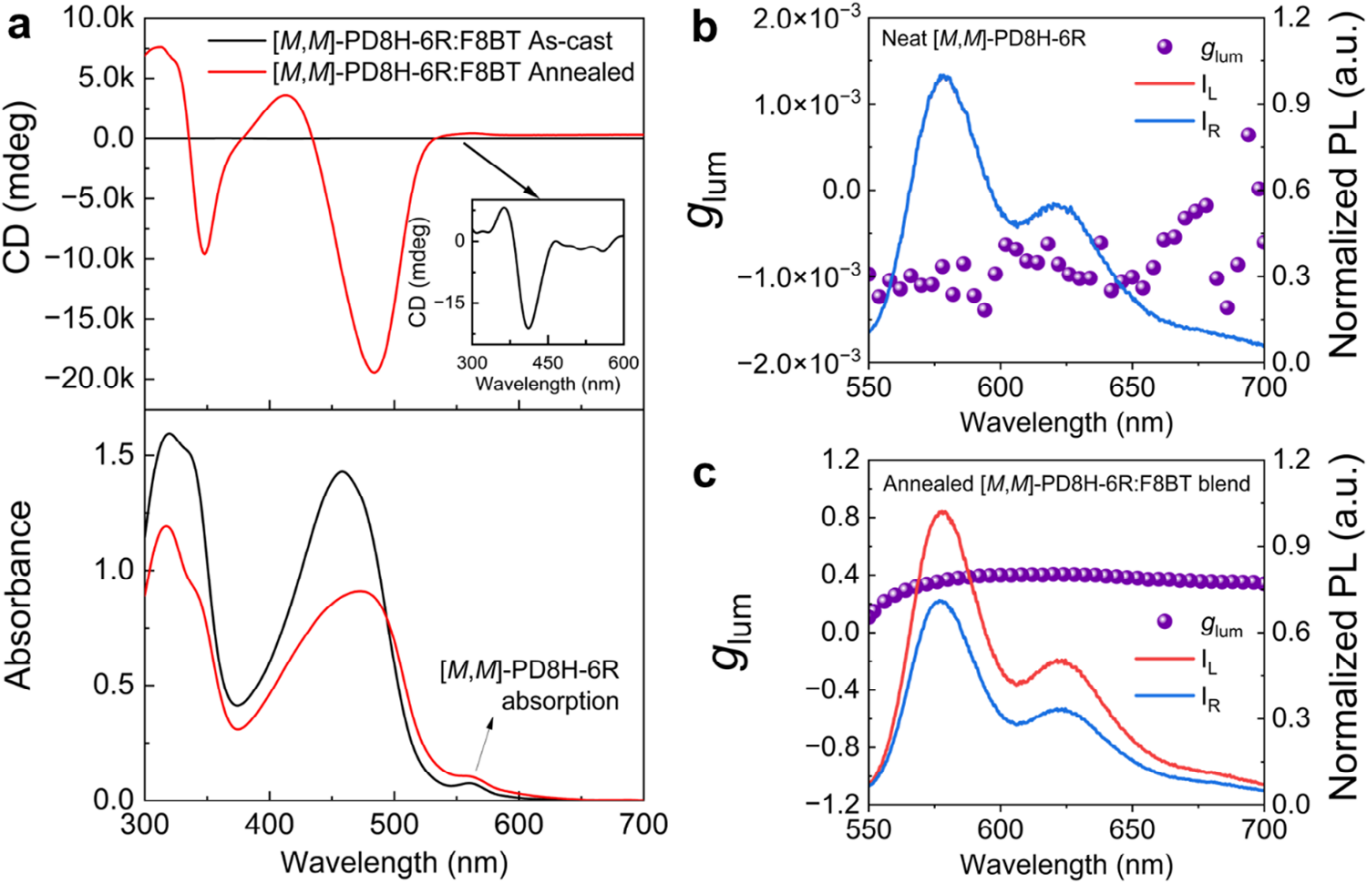
Chiroptical responses of the F8BT chiral host and CPL signals of the double helicene emitter. a) Circular dichroism and absorption profile of as‐cast and annealed [*M*,*M*]‐PD8H‐6R:F8BT blend thin films; the inset is the zoomed CD spectrum for the as‐cast film. b) Unamplified CPL spectra of neat [*M*,*M*]‐PD8H‐6R. c) Amplified [*M*,*M*]‐PD8H‐6R CPL signals in an annealed [*M*,*M*]‐PD8H‐6R:F8BT blend. The annealing temperature for the blend films is 170 °C. CPL was collected here using a JASCO CPL‐300 spectrometer with λ_ex_ = 475 nm.

Since [*M*,*M*]‐ and [*P*,*P*]‐PD8H‐6R demonstrate mirror chiroptical responses (Figure S3, Supporting Information), we focus on the [*M*,*M*]‐enantiomer for the rest of the discussion. An [*M*,*M*]‐PD8H‐6R thin film (≈80 nm) experimentally exhibits a circular dichroism (CD) of ≈−172 mdeg at λ = 565 nm with a *g*‐factor of −2.7 × 10^−3^ (Figure S4, Supporting Information), which is calculated by replacing *I*_L/R_ with left‐ and right‐handed absorbances (*A*_L/R_) in Equation (2). Neat F8BT is CD silent;^[^ ^25^, ^28^ ^]^ however, blending 10 wt.% [*M*,*M*]‐PD8H‐6R into the F8BT polymer and followed with an established thermal annealing process triggers F8BT chirality,^[^ ^5^, ^28^ ^]^ which then creates a chiral environment for the double helicene. The absorption profile of the blend shows absorption features of both the polymer and the double helicene (**Figure** **2**a). The polymer absorption is red‐shifted, and the corresponding fluorene‐to‐BT charge transfer transition peak broadens (Figure 2a). This indicates that the electronic states of the polymer undergo a Davydov splitting,^[^ ^29^ ^]^ giving rise to the bisignate CD bands, consistent with a number of previous reports.^[^ ^28^, ^30^, ^31^ ^]^ A F8BT CD signal at λ = 484 nm emerges when the blend was annealed beyond the glass transition temperature of the polymer (*T*_g_ > 120 °C). The strongest CD signal ≈−19 455 mdeg (*g*_abs_ = −0.77) was observed when the blend film was heated up to 170 °C, confirming the formation of a chiral environment for the double helicene emitter^[^ ^5^, ^25^ ^]^ (Figure S5, Supporting Information). Further increasing the annealing temperature toward the melting temperature (*T*_m_ > 240 °C) leads to the randomization of the polymer chains, which destroys the chiral environments.

We first performed CPL characterization for as‐cast and annealed blend films by exciting the polymer absorption band at λ = 475 nm. Without the annealing process, the chiral environments are not formed, thus [*M*,*M*]‐PD8H‐6R emission from the as‐cast blend film demonstrates unamplified weak CPL signal with a *g*_PL_ of ≈−2.1 × 10^−3^ at λ = 578 nm (Figure S6, Supporting Information), similar to its neat film (Figure 2b). However, after the chiral environments are induced by the annealing process, the resulting CPL signal from [*M*,*M*]‐PD8H‐6R exhibits a *g*_PL_ of ≈+0.36 at its main emission peak at λ = 578 nm and a larger *g*_PL,max_ of ≈+0.40 at its shoulder emission peak (λ = 623 nm), demonstrating one of the highest *g*_PL_ values from organic small‐molecule emitters. The increase in CPL signal is highly correlated with the formation of an F8BT chiral environment upon annealing (Figures S7 and S8, Supporting Information). Compared with dilute solution‐state *g*_PL_, the chiral host environment boosts the |*g*_PL_| signals by ≈133 times from ≈3.0 × 10^−3^ to ≈0.40 (Figure 2b,c), while simultaneously increasing the helicene PLQY from ≈13% in its neat form to ≈47% in the annealed blend form (Table S4, Supporting Information). Interestingly, we observed that the amplified CPL exhibits a sign inversion compared to the unamplified CPL signals for both [*M*,*M*]‐ and [*P*,*P*]‐PD8H‐6R enantiomers (Figure S9, Supporting Information). Specifically, the [*M*,*M*]‐enantiomer, which typically emits right‐handed CPL,^[^ ^24^ ^]^ is found to emit left‐handed CPL when embedded in the chiral F8BT environment. A 48‐sample statistical analysis (Figure S10 and Table S5, Supporting Information) also confirms that the inversion is not observed in just a single instance; our thin‐film deposition and processing methods are robust and highly reproducible. This inverted CPL signal remains constant regardless of film thickness ranging from 30 to 1200 nm and regardless of sample orientation or measurement geometry; this was verified with two commercially available CPL spectrometers (JASCO CPL‐300 uses a 180° geometry; Olis CPL spectrometer uses a 90° geometry, see Methods and Figure S11, Supporting Information for a detailed comparison of the measurement geometry). The absence of Bragg reflection features in reflection circular dichroism also indicates that there is negligible selective reflection of circularly polarized light in these films, which is consistent with prior studies using Mueller Matrix Spectroscopy^[^ ^28^ ^]^ (Figure S12, Supporting Information). Therefore, this sign inversion, which deviates from previously reported cases,^[^ ^18^, ^19^, ^20^, ^32^, ^33^ ^]^ suggests a fundamental alteration in the photophysical properties of the emitter, rather than a mere amplification of the existing signal.^[^ ^18^, ^19^, ^20^ ^]^

**Figure 3:**
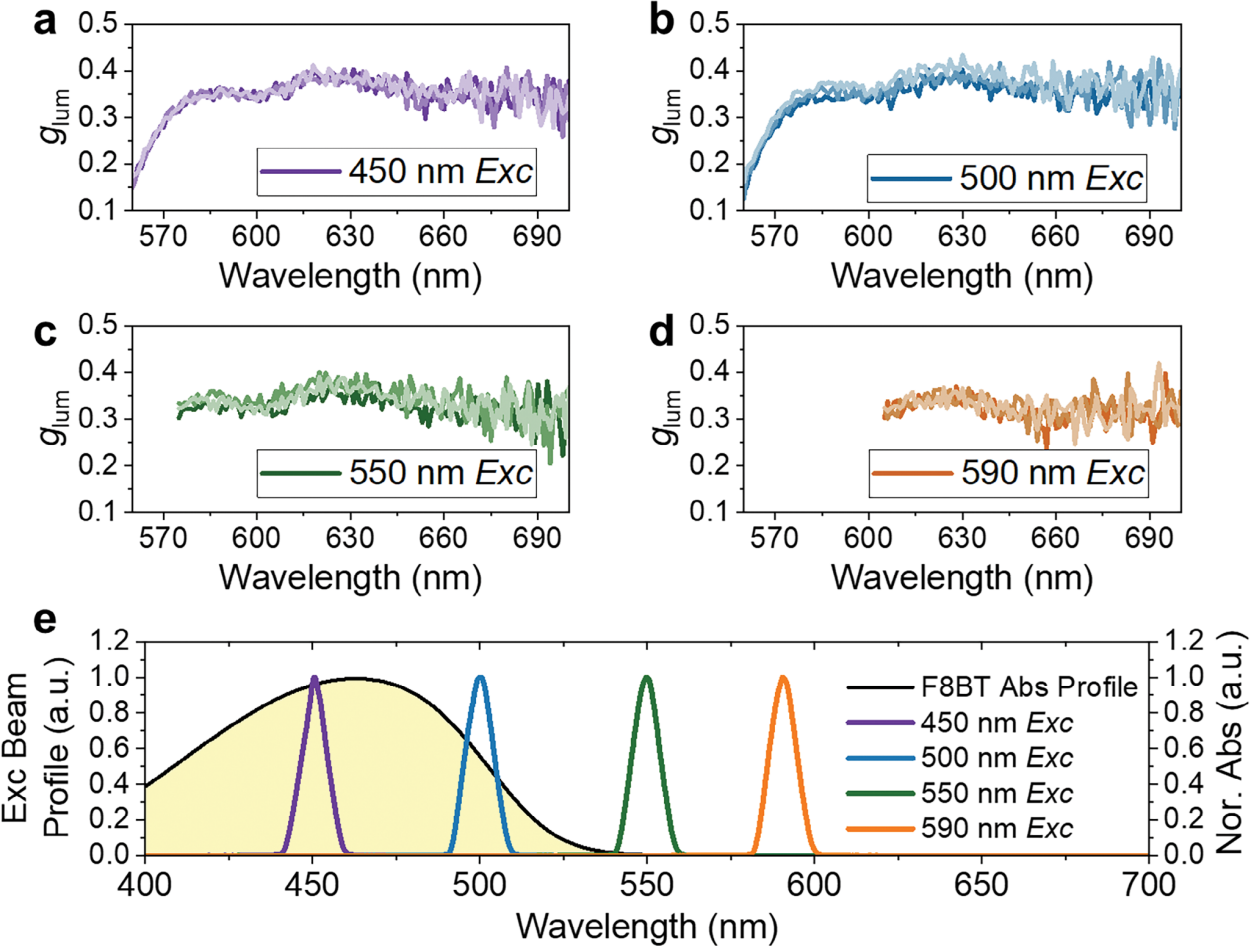
Amplified chiral emission of the [*M*,*M*]‐PD8H‐6R:F8BT blend. CPL spectra collected at different excitation wavelengths: a) 450 nm; b) 500 nm; c) 550 nm; d) 590 nm. Each plot gives 3 unaveraged unfiltered scans. CPL was collected here using an Olis CPL spectrometer with an external light source with 10 nm bandpass filters. e) Beam profile of all excitation beams used. 590 nm bandpass filter blocks 200–576 nm, 604–1200 nm with OD_abs_ > 5, ensuring zero overlap with F8BT absorption profile.

To investigate the role of the chiral polymeric environment and the possible origins for the amplified and inverted CPL signals from the molecular emitter, we compared the CPL spectra recorded under different excitation wavelengths (**Figure** **3**a–d). For the best contrast between left‐ and right‐handed PL signals, we examined [*M*,*M*]‐PD8H‐6R:F8BT blend thin films that were annealed at 170 °C, and chose four excitation wavelengths: 450, 500, 550, and 590 nm. According to the absorption profile (Figure 3e), F8BT is strongly absorbing at 450 nm, which allows strong FRET from F8BT to [*M*,*M*]‐PD8H‐6R; on the other hand, for excitations at 532 nm and at the absorption edge of 550 nm, the F8BT absorption as well as FRET is strongly suppressed. For the 590 nm excitation, a bandpass cut‐off of OD_abs_ > 5 at 576/604 nm (Figure 3e) ensures F8BT is not excited. In this case, the CPL emitted from the thin films solely originates from the helicene's absorption with negligible contribution from any FRET process. For all CPL spectra recorded under the different excitations, the data show an inverted CPL signal and a consistent *g*_lum_ value of ≈+0.35 (±0.01) at λ_em_ = 580 nm and +0.40 (±0.02) λ_em_ = 623 nm. This points to the important aspect that the *CPL amplification effect is not directly dependent on FRET*. These experiments were also conducted in parallel using the circularly polarized luminescence excitation (CPLE) mode (see Methods). The *g*_lum_ probed at two emission peaks both show negligible changes (Figure S13, Supporting Information). To further confirm that the inverted and amplified signal does not rely on FRET, we replaced F8BT with a polyfluorene homo polymer – poly(9,9‐dioctylfluorenyl‐2,7‐diyl) (PFO); PFO performs similarly to F8BT in forming chiral environments^[^ ^34^ ^]^ and has a wider excitation window that allows us to only excite the emitter. Even when exciting at 550 nm (≈80 nm from the PFO absorption edge at 470 nm), the *g*_lum_ probed for [*M*,*M*]‐PD8H‐6R remains inverted and at the same order of magnitude (*g* ≈+ 0.10, Figure S14, Supporting Information), consistent with our observations in F8BT:[*M*,*M*]‐PD8H‐6R blends). Thus, these results provide additional evidence that the observed CPL amplification effect does not rely on any energy transfer process.

We also ruled out significant morphological changes that might be associated with the amplified chiroptical responses by performing different morphological characterization measurements. We performed Atomic Force Microscopy (AFM) imaging on F8BT polymer and blends (Figure S15, Supporting Information). As‐cast neat polymer and blend both demonstrate extremely low roughness (*R*_mean‐square_ <0.5 nm), demonstrating high isotropy and featureless amplitude and phase profiles in AFM images. After annealing, we could only observe a granular nanoscale morphology, negating crystalline or cholesteric liquid‐crystalline morphology. F8BT's weakly crystalline and highly isotropic features are also well‐characterized by grazing incidence wide‐angle scattering (GIWAXS) measurements. Only a broad arc can be observed (*q* ≈ 15 nm⁻¹, corresponding to *d* ≈4.18 Å) in a number of previously reported 2D GIWAXS images.^[^ ^5^, ^25^ ^]^ Therefore, 1D azimuthally integrated profiles were used here as a better description of GIWAXS results for as‐cast and annealed F8BT and PD8H‐6R:F8BT blend films (Figure S16, Supporting Information). By comparing the *π–π* stacking peaks in out‐of‐plane profiles and the in‐plane profiles, it can be concluded that the F8BT films remain highly isotropic. Additionally, neat and blended films show almost identical *π–π* stacking distances of *d* = 4.18 Å before and after thermal annealing. By combining AFM and GIWAXS characterizations, we ruled out that the CPL amplification is caused by any form of significant morphological changes. It is noted that while these characterization techniques are particularly effective for analyzing crystalline structures, they exhibit substantial limitations in evaluating amorphous chiral structures, which, in fact, constitute the majority in F8BT and blend thin films.

Having established that there occur negligible energy transfer processes and packing modifications in the blends, we next focused on the electronic‐structure changes within the blend systems and investigated the origin of the amplified and inverted CPL signals. Since such intense CPL signals and large *g*_PL_ values cannot be usually obtained via single‐molecule emission, we took a stepwise approach and calculated the *g*‐factor changes at the time‐dependent density functional theory (TD‐DFT) level in going from an isolated double helicene molecule to a complex aggregate system involving the F8BT chiral environment (see Methods).

For a single [*M*,*M*]‐PD8H‐6R molecule, our TD‐DFT calculations yield a rotatory strength of −114 × 10^−40^ erg∙esu∙cm∙G^−1^, corresponding to a *g*‐factor of −3.2 × 10^−3^, which is in good agreement with the experimental results (Table S6, Supporting Information).^[^ ^24^ ^]^ When extending these calculations to helicene dimers (Figure S17 and Table S6, Supporting Information), we observe that the dipole–dipole interactions between the two helicene molecules remain weak, primarily due to steric hindrance among the branched side chains, a feature that prevents any amplification of the *g*‐factors. Whether we consider an optimized dimer structure or additionally involve a ±30° intermolecular rotation, the *g*‐factors of these dimers are all negative and remain of the same order of magnitude as that of the single molecule (≈10^−3^). These results indicate that the intermolecular interactions between the helicene molecules are not the source of the observed CPL *g*‐factor sign inversion. In contrast, stacks of F8BT chromophores exhibit strong dipole–dipole interactions due to a short π‐π stacking distance of 4.18 Å, as identified by GIWAXS measurements (Figure S16, Supporting Information). By introducing helical intermolecular rotations to the π‐stacked systems,^[^ ^30^, ^31^ ^]^ the exciton coupling among these oriented F8BT chromophores generates strong intermolecular chirality. Our results also demonstrate that when F8BT molecules adopt helical in‐plane orientations, they exhibit a significant enhancement in rotatory strength and a high *g*‐factor (Figures S18, S19, and Table S7, Supporting Information). However, considering that there is negligible contribution from the FRET process, the amplified CPL from the helicene molecules cannot be directly linked with the main CD absorption band at λ = 475 nm.

We noticed that the broadening of the absorption profile of F8BT also brings about a positive CD shoulder at λ = 570 nm (Figure 1). This shoulder is anticipated to correspond to a dark lower energy state, which overlaps with the [*M*,*M*]‐PD8H‐6R absorption profile. This is confirmed by our TD‐DFT calculations: When forming F8BT dimers, the S_1_ state of the monomer splits into two states, a bright S_2_ state (*f* = 1.38, *R* = −69 × 10^−40^ erg∙esu∙cm∙G^−1^) and a much less absorbing S_1_ state (*f* = 0.05, *R* = +623 × 10^−40^ erg∙esu∙cm∙G^−1^; Figure S20 and Table S8, Supporting Information). The negative *R*(S_2_) and positive *R*(S_1_) values correspond to the negative main peak and positive shoulder peak in the F8BT CD profile, respectively (Figure 1). We also observed intensity borrowing^[^ ^35^, ^36^ ^]^ in the blend films in absorption measurements, where after annealing, the absorption peak that corresponds to [*M*,*M*]‐PD8H‐6R increases while the F8BT peak drops, features consistent with the appearance of excited‐state hybridization (See Figure 2a; ‘Further Discussion’ Section in Supporting Information). We then surmised that, if these F8BT low‐energy excited states can electronically hybridize with helicene excited states of a similar energy, the helicene electronic structure could be significantly affected by these F8BT excited states, potentially aligning its CPL amplitude and sign to that of the F8BT environment.

**Figure 4:**
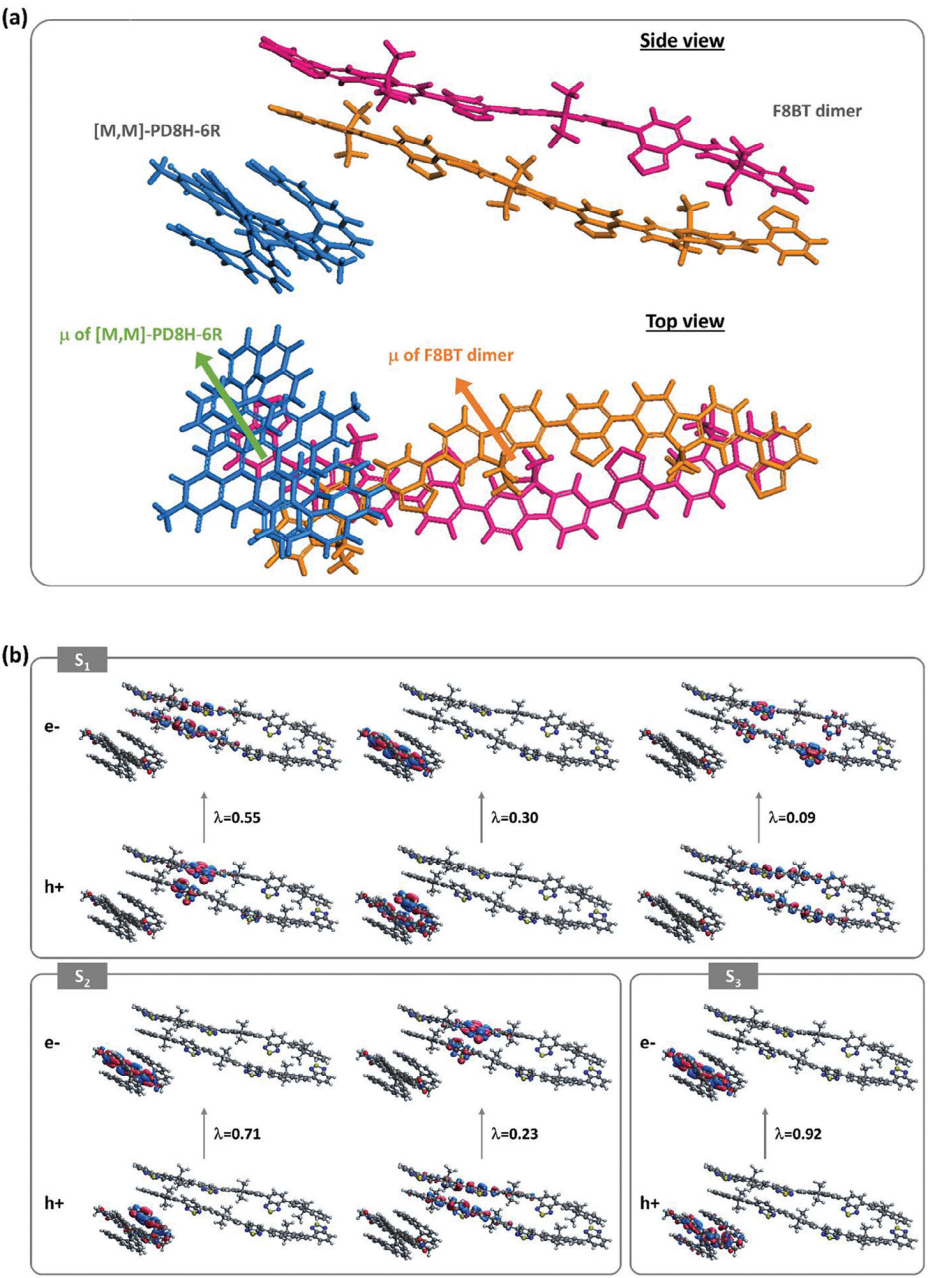
TD‐DFT results for an F8BT dimer‐[*M*,*M*]‐PD8H‐6R complex. a) Side and top views of the complex: The electric transition dipole moments (***μ***) of helicene and dimer are aligned in parallel. b) Electron and hole natural transition orbitals (NTO) in excited states S_1_‐S_3_ of the complex (λ indicates the fraction of the specific hole–electron transition to the excitation).

To verify this hypothesis, we performed TD‐DFT calculations on a blend system consisting of a geometry‐optimized helicene molecule placed next to a geometry‐optimized F8BT dimer. When the directions of the electric transition dipole moments of the [*M*,*M*]‐PD8H‐6R molecule and F8BT dimer align in parallel, it turns out that the helicene‐F8BT complex shows inverted and amplified positive *g*‐factors of +0.243, +0.029, and +0.002 for the S_1_, S_2_, and S_3_ states, respectively (Table 1, **Figure** **4**; Figure S21, Table S9, Supporting Information). We note that the lowest six singlet states of the complex are very close in energy due to the (near) degeneracy of the lowest three excited states of the helicene and of the lowest two excited states of the dimer; it should be kept in mind that the helicene molecule itself exhibits negative *g*‐factors for its lowest three singlet states (Table 1; Table S6, Supporting Information).

By investigating the nature of the electronic transitions that contribute to each excited state in the helicene‐F8BT complex, we find that S_1_ and S_2_ are *hybrid* states and exhibit amplified inverted (positive) g‐factors. The S_1_ wavefunction has a 30% contribution from the helicene and 64% from the F8BT dimer, while the S_2_ wavefunction is 71% helicene and 23% F8BT dimer. In contrast, S_3_ is an excited state with almost sole contributions from the helicene (92%); in spite of this, it displays an inverted (positive) g‐factor, which provides direct evidence of the intrinsic electronic‐structure changes induced in the helicene molecule through the interaction with the F8BT dimer. On the other hand, the S_4_ to S_6_ states have negative *g* values (Figure S20, Supporting Information); in the S_4_ and S_6_ states, the holes are distributed on both helicene and F8BT dimer and electrons are mostly localized on the helicene PDI unit, while in the S_5_ state holes and electrons are located on F8BT.

Introducing mutual rotations of the electric transition dipoles between [*M*,*M*]‐PD8H‐6R and the F8BT dimer has an impact on the amplitude of the *g*‐factors (see Figures S22–S25, Tables S10 and S11, Supporting Information); however, the *g*‐factor signs associated with the lowest‐energy excited states remain unchanged. We also note that for particular mutual angles of electric transition dipoles between [*M*,*M*]‐PD8H‐6R and the F8BT dimer, transitions that are even highly localized in helicene structures (> 90%), such as S_0_ → S_3_ in parallel position (Table 1) and S_0_ → S_2_ in +60° (Table S11, Supporting Information) demonstrate sign‐inverted *R* and *g*‐factors. This further confirms that through excited‐state hybridization, helicene's excited‐state chiroptical response could revert its handedness without flipping the ground‐state molecular geometries. Although detailed molecular dynamics investigations would be needed to fully describe the blend system, our DFT calculations based on model molecular complexes show that the amplification and sign inversion of the *g*‐factor of the double helicene embedded in a chiral environment can originate from an intrinsic electronic‐structure change that is associated with helicene‐polymer excited‐state wavefunction hybridization. More specifically, it is the alignment between the electric dipoles of the helicene excited state and the chiral ‘dark’ state of the chiral host environment that is the source of the significant CPL signal amplification. This origin is fundamentally different from the requirements usually considered in energy transfer processes.

## 3 Conclusion

In summary, we achieved a record‐high chiral emission *g*‐factor of ≈0.40 from a small‐molecule helicene emitter by embedding it into a chiral polymeric environment. Beyond the amplitude amplification of the *g*‐factor, there occurs an associated *g*‐factor sign inversion. Through photophysical characterizations, we can rule out that factors such as energy transfer or morphological changes in the solid‐state thin films play a role and attribute the inverted amplified chiral emission to electronic‐structure changes of the emitters in the blends. We find that the rotatory strength of the molecular emitter can be dramatically enhanced and its sign inverted to align with that of the chiral environment. Such rotatory strength and resulting CPL signal amplification originate from the hybridization of the lowest excited states between the chiral emitters and their chiral polymeric environment. From a spectroscopic perspective, it requires the emission spectra of the chiral emitters to overlap with the absorption of “chiral dark states” in chiral environments (sensitizers), which is fundamentally different from the requirements of FRET processes.

We believe our findings offer new paths for the design of chiral emissive systems that are not limited by the constraints related to energy transfer processes. Our results regarding the energy alignment of the lowest excited states in the emitters and the chiral host environment provide novel strategies for strongly circularly polarized emissive systems.

## 4 Experimental Section

### Materials

F8BT and PFO were purchased from Lumtec. Ltd and used without further purification. The double helicenes PD8H‐6R were synthesized using the reported method.^[^ ^24^ ^]^

### Solution Preparation and Thin Film Deposition

The double helicenes and F8BT were dissolved in toluene to a concentration of 15 mg mL^−1^ and blended to form a 10 wt.% solution. Thin films were spin‐coated at a speed of 1200 rpm to give a ≈120 nm film and deposited on clean fused silica substrates. The cleaning process for all substrates involved rinsing in an ultrasonic bath with acetone, isopropyl alcohol (IPA), Hellmanex III (Sigma–Aldrich), and deionized water for 15 min. These were followed by a UV‐ozone treatment for 10 min before spin‐coating. The thickness of the thin films was controlled by spin speed and monitored using a Dektak XT surface profiler.

### Photophysical and Morphological Characterization

CD spectra and absorption spectra were collected using a Chirascan (Applied Photophysics) spectrometer at Linköping University and an Olis DSM172 CD spectrometer at Max Planck Institute of Microstructure Physics. The Olis DSM 172 CD spectrometer allows PMT to be positioned in 90° geometry for selective reflection measurement (Figure S12, Supporting Information). CPL measurements have been conducted using both the Olis all‐in‐one CPL spectrometer at the Max Planck Institute of Microstructure Physics and a JASCO CPL‐300 spectrometer at the University of Copenhagen. Mounted LED Thorlabs M450LP2, M505L5, M565L3^n^, and M590L4 were used as excitation light sources with Olis CPL spectrometer with corresponding bandpass filters FBH450‐10, FBH500‐10, FBH550‐10, and FBH590‐10. JASCO CPL‐300 uses a 180° geometry while the Olis DSM 172 CPL spectrometer uses a 90° geometry (Figure S11, Supporting Information). The dissymmetry factor *g*_abs_ was calculated following the equation *g* = (*A*_L_ – *A*_R_)/*A*, |*g*| ≤ 2. Grazing incidence wide‐angle scattering (GIWAXS) data were recorded at the NCD‐SWEET beamline (ALBA synchrotron in Cerdanyola del Vallès, Spain) with a monochromatic (λ = 0.9603 Å) X‐ray beam of 80 × 30 µm^2^ [H × V], using a Si (111) channel cut monochromator. The scattered signal was recorded using a Rayonix LX255‐HS area detector placed at 241.1 mm from the sample position. The reciprocal q‐space and sample‐to‐detector distance were calculated using Cr_2_O_3_ as a calibrant. A near‐critical angle of incidence of 0.12° was used to maximize the thin film signal, and the collected 2D images were azimuthally integrated using PyFAI.^[^ ^37^ ^]^ AFM imaging for F8BT and blend films was performed using a non‐contact mode AFM (FX40, Park Systems). PLQY of F8BT and blend samples were measured with a 375 nm excitation, using a Newton 971 EMCCD in a Kymera 328i spectrograph system with an integrating sphere and a method described by Ahn et al.^[^ ^38^ ^]^

### DFT Calculations

The ground‐state geometries of an isolated [*M*,*M*]‐PD8H‐6R molecule and an F8BT chain consisting of three repeat units were optimized at the LC‐ωHPBE/6‐31G^**^ level of theory with GD3BJ dispersion corrections; here, all side chains were replaced with hydrogen atoms. The [*M*,*M*]‐PD8H‐6R dimer and F8BT dimer were optimized using the GFN2‐xTB method^[^ ^39^ ^]^ in the xTB software;^[^ ^40^ ^]^ here, the full side chains were considered for the geometry optimizations but replaced with hydrogen atoms for the subsequent TD‐DFT calculations. TD‐DFT calculations of the molecules, dimers, and complexes were carried out at the LC‐ωHPBE/6‐31G^**^ level and considering a dielectric constant of ε = 2.38 to account for the effect of a toluene medium (unless otherwise noted). The electric and magnetic transition dipole moments were given in CGS‐Gaussian units. The TD‐DFT and DFT calculations were performed with the Gaussian 16 code.^[^ ^41^ ^]^

## Conflict of Interest

The authors declare no conflict of interest.

## Author Contributions

L.W. and E.C. contributed equally to this work. L.W. and F.G. conceived the project. L.W. performed all spectroscopic measurements. R.Z. performed GIWAXS measurement. E.C., C.V., and J.‐L.B. performed the DFT calculations and provided the theoretical analysis. L.W. wrote the manuscript. All authors contributed to the discussion of the results and the editing of the manuscript.

## Supporting information



Supporting Information

## Data Availability

The data that support the findings of this study are available from the corresponding author upon reasonable request.
