# Supplementary material for: Giant Circularly Polarized Luminescence Driven by Excited‐State Hybridization Between Molecular Emitters and Chiral Environments
Source: Adv Mater. 2025 Jul 16;37(40):2506941. doi: 10.1002/adma.202506941 (PMC12510273; doi:10.1002/adma.202506941)
Supplement: Supplementary file 1 — Supporting Information [file ADMA-37-2506941-s001.docx]

Supplementary Information

Giant circularly polarized luminescence driven by excited-state hybridization between molecular emitters and chiral environments

Li Wan^1,2,7*^, Eunkyung Cho^3,4,7^, Rui Zhang^1^, Theis Brock Nannestad^5^, Zhaohui Wang^6^, Jean-Luc Brédas^4^, Veaceslav Coropceanu^4*^, Feng Gao^1^

^1^Department of Physics, Chemistry and Biology (IFM), Linköping University, Linköping, 58432, Sweden

^2^Max Planck Institute of Microstructure Physics, Halle, 06120, Germany

^3^Divison of Energy and Environmental Technology, DGIST, Daegu, 42988, Republic of Korea

^4^Department of Chemistry and Biochemistry, The University of Arizona, Tucson, Arizona, 85721-0041, USA

^5^Department of Chemistry, University of Copenhagen, Copenhagen, 2100, Denmark

^6^Key Laboratory of Organic Optoelectronics and Molecular Engineering, Department of Chemistry, Tsinghua University, Beijing, 100084, China

^7^These authors contributed equally: Li Wan, Eunkyung Cho

*li.wan@mpi-halle.mpg.de, *coropceanu@arizona.edu

**Table S1** Comparisons of different approaches for amplifying circularly polarized luminescence for chiral organic small molecules. Extrinsic amplification methods such as those related to the use of external magnetic field, optical filters, or liquid crystal layers are not included in this table.

| Material | Amplification Mechanisms | *λ*_em_ (nm) | *g*_lum,_ Unamplified | *g*_lum,_  Amplified | Amplification Factor | Comments |
| --- | --- | --- | --- | --- | --- | --- |
| *R*-BINDPA^1^ | Triplet–Triplet annihilation-based photon upconversion (TTA-UC) using a PtOEP sensitizer | 450 | 2 × 10^–4^ | 4 × 10^–3^ | 20 | Solution-state |
| *R*-TP^2^ | TTA-UC using TADF a sensitizer | 380 | 3.1 × 10^−3^ | 9.2 × 10^−3^ | 3 | TTA-UC in solution, g_lum_ further amplified to 0.19 using a liquid crystal matrix |
| oxa[7]H^3^ | Förster resonance energy transfer (FRET) amplified CPL | 540 | 3 × 10^−4^ | 0.15 (@ 560 nm) | 500 | *g*_lum_ at emission wavelength < 0.1 |
| **This work** | **Excited-state hybridization** | **580** | **−3.0 × 10^−3^** | **+0.40 (@ 610 nm)** | **133** | *g*_lum_ at emission wavelength = 0.36 |


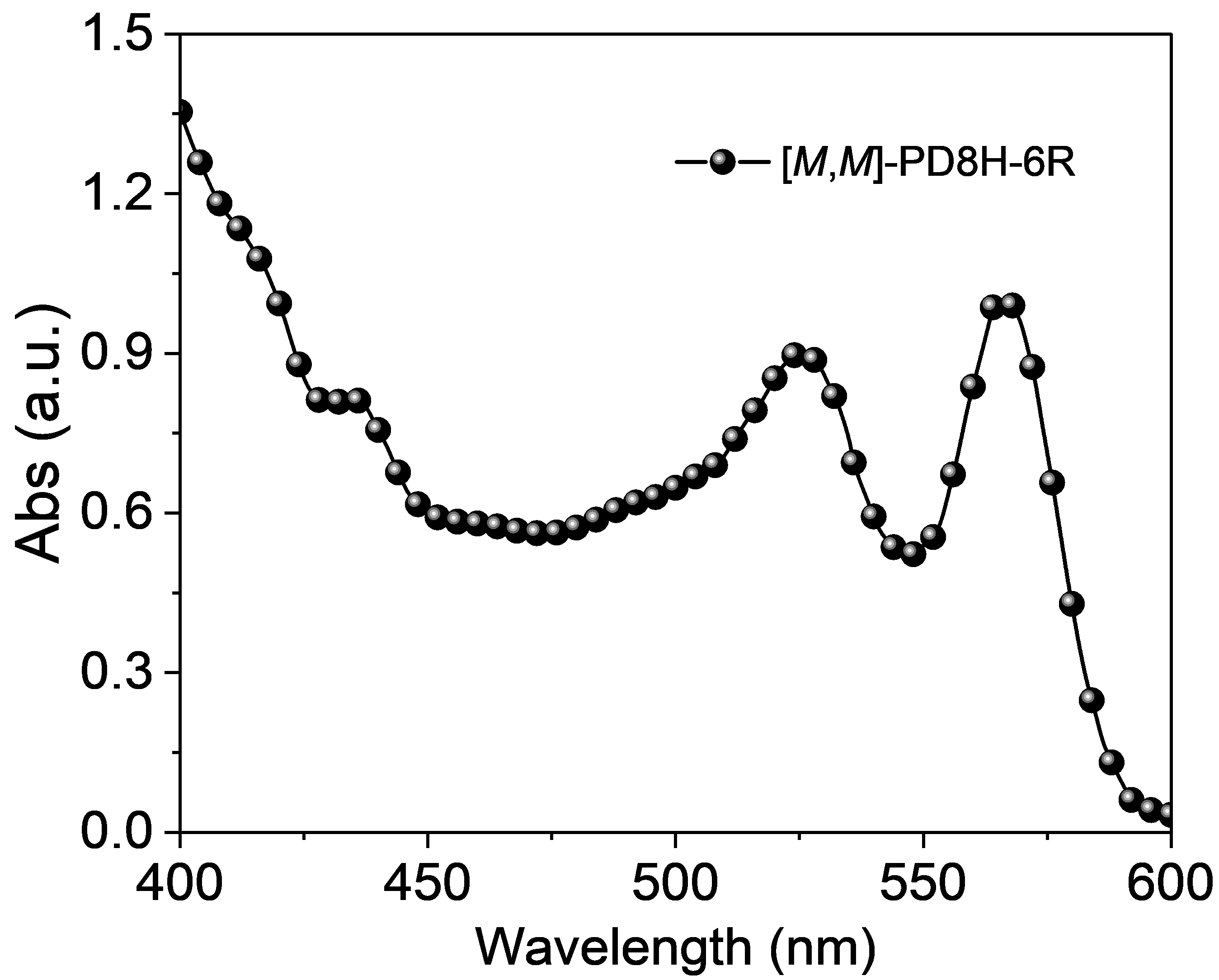


**Fig. S1** Absorption profile of [*M*,*M*]-PD8H-6R in a dilute chloroform solution (1 × 10^–5^ M).


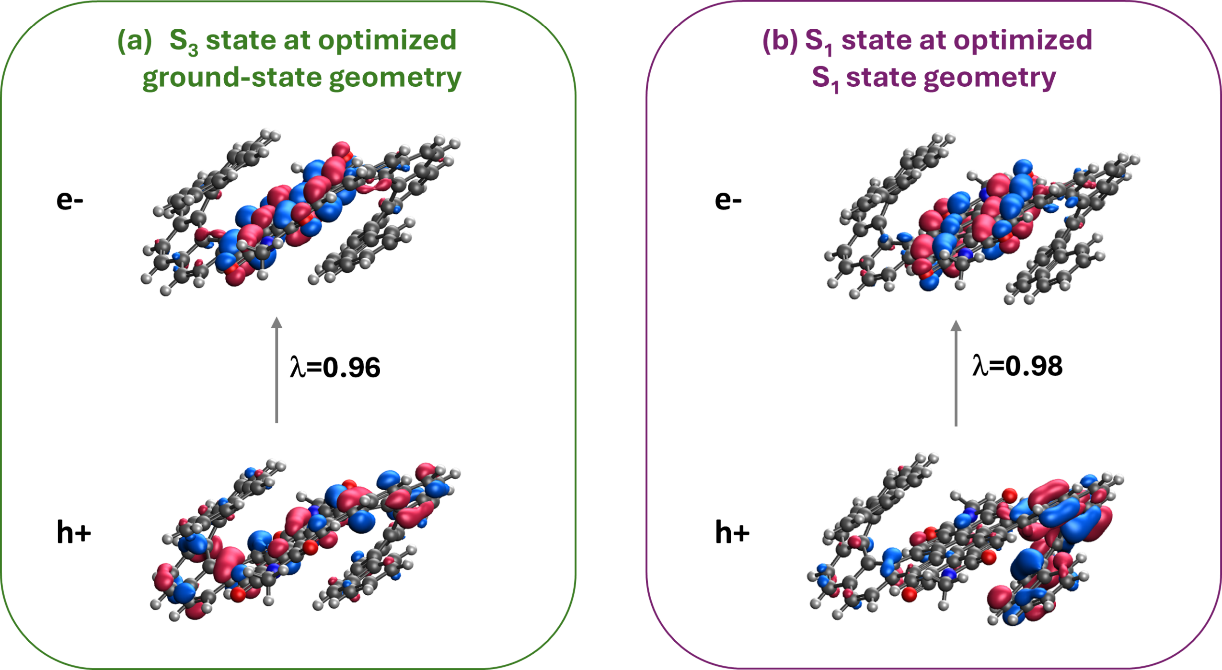


**Fig. S2** Natural Transition Orbitals (NTOs) in a single [*M*,*M*]-PD8H-6R molecule: (a) in the S_3_ state at optimized ground-state geometry (see Table S2) and (b) in the S_1_ state at optimized S_1_ state geometry (see Table S3). Both transitions have charge-transfer (CT) character with electron transfer from a helicene segment to the PDI moiety.

**Table S2** Calculated absorption and CD properties of a single [*M*,*M*]-PD8H-6R molecule.

|  | E  (eV) | E  (nm) | *f* | \|***μ***\|  (10^−20^ esu cm) | \|***m***\|  (10^−20^ erg G^−1^) | Cos(*θ*^b^) | *R*  (10^−40^ erg  esu cm G^−1^) | *g* |
| --- | --- | --- | --- | --- | --- | --- | --- | --- |
| S_0_ → S_1_ | 2.23 | 556 | 0.016 | 138 | 0.15 | -0.97 | -20 | -0.004 |
| S_0_ → S_2_ | 2.24 | 555 | 0.000 | 14 | 0.42 | -0.08 | 0 | -0.009 |
| **S_0_ → S_3_** | **2.27** | **547** | **0.108** | **354** | **1.06** | **-0.33** | **-126** | **-0.004** |
| S_0_ → S_4_ | 2.32 | 534 | 0.020 | 152 | 0.54 | -1.00 | -82 | -0.014 |
| S_0_ → S_5_ | 2.45 | 506 | 0.031 | 183 | 0.73 | 0.68 | 91 | 0.011 |
| S_0_ → S_6_ | 2.56 | 484 | 0.097 | 316 | 0.27 | 1.00 | 84 | 0.003 |
| S_0_ → S_7_ | 2.87 | 431 | 0.003 | 53 | 0.74 | -0.85 | -34 | -0.047 |
| S_0_ → S_8_ | 2.90 | 428 | 0.089 | 285 | 2.86 | -0.71 | -575 | -0.028 |
| S_0_ → S_9_ | 2.94 | 422 | 0.006 | 72 | 0.85 | 1.00 | 61 | 0.047 |
| S_0_ → S_10_ | 2.96 | 419 | 0.002 | 40 | 1.33 | -1.00 | -53 | -0.133 |

Geometry optimizations at the LC-ωHPBE/6-31G** level with GD3BJ dispersion corrections; TDDFT calculations at the LC-ωHPBE/6-31G** level with consideration of a dielectric constant of ε=4.3 to account for the effect of a chloroform medium. The energy state marked in orange corresponds to the 565 nm absorption in the experimental data.

**Table S3** Calculated CPL properties of a single [*M*,*M*]-PD8H-6R molecule.

|  | E  (eV) | E  (nm) | *f* | \|***μ***\|  (10^−20^ esu cm) | \|***m***\|  (10^−20^ erg G^−1^) | Cos(*θ*^b^) | *R*  (10^−40^ erg  esu cm G^−1^) | *g* |
| --- | --- | --- | --- | --- | --- | --- | --- | --- |
| S_0_ → S_1_ | 2.12 | 584 | 0.066 | 287 | 0.79 | -0.47 | -106 | -0.005 |
| S_0_ → S_2_ | 2.14 | 579 | 0.037 | 215 | 0.52 | -0.31 | -35 | -0.003 |
| S_0_ → S_3_ | 2.19 | 567 | 0.062 | 274 | 0.66 | -0.13 | -24 | -0.001 |

Geometry optimizations in the S_1_ excited state at the LC-ωHPBE/6-31G** level with GD3BJ dispersion corrections; TDDFT calculations at the LC-ωHPBE/6-31G** level with consideration of a dielectric constant of ε=4.3 to account for the effect of a chloroform medium. The experimental absorption data (Fig. S1) and CD/CPL data of [*M*,*M*]-PD8H-6R in dilute solution are very well reproduced^4^.


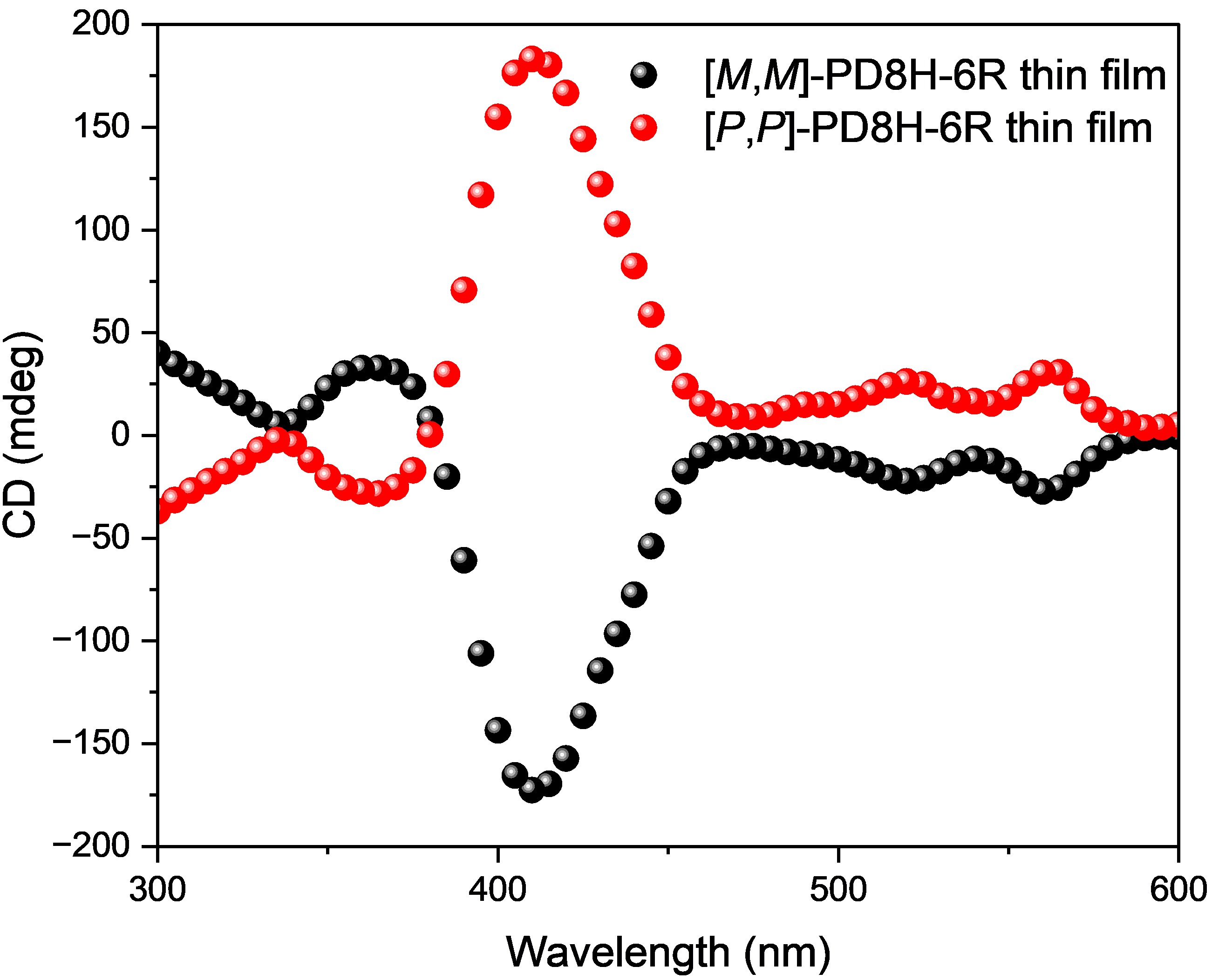


**Fig. S3** [*M*,*M*]- and [*P*,*P*]-PD8H-6R CD profiles.


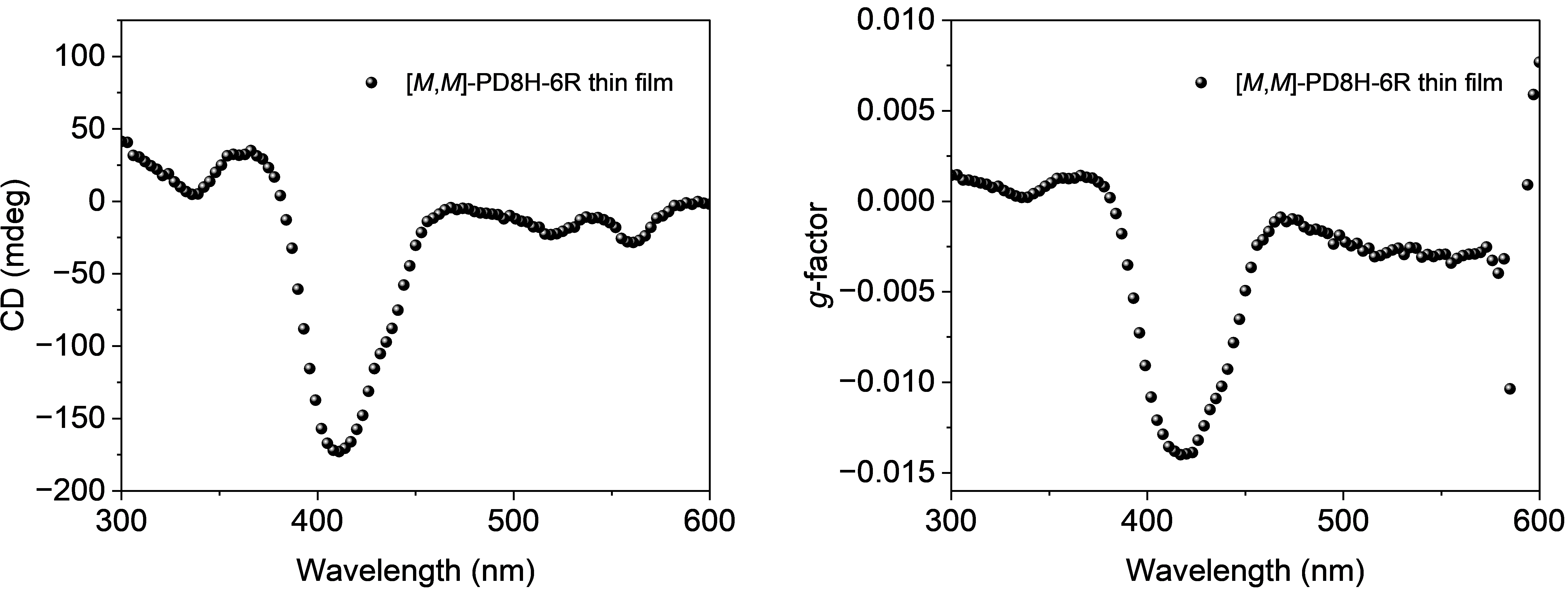


**Fig. S4** CD profile of neat [*M*,*M*]-PD8H-6R thin films. The thin-film data are consistent with the CD profile of [*M*,*M*]-PD8H-6R in dilute solution, indicating negligible intermolecular excitonic coupling among the helicene emitters. *g*_abs_(565 nm) = 2.7 × 10^–3^.


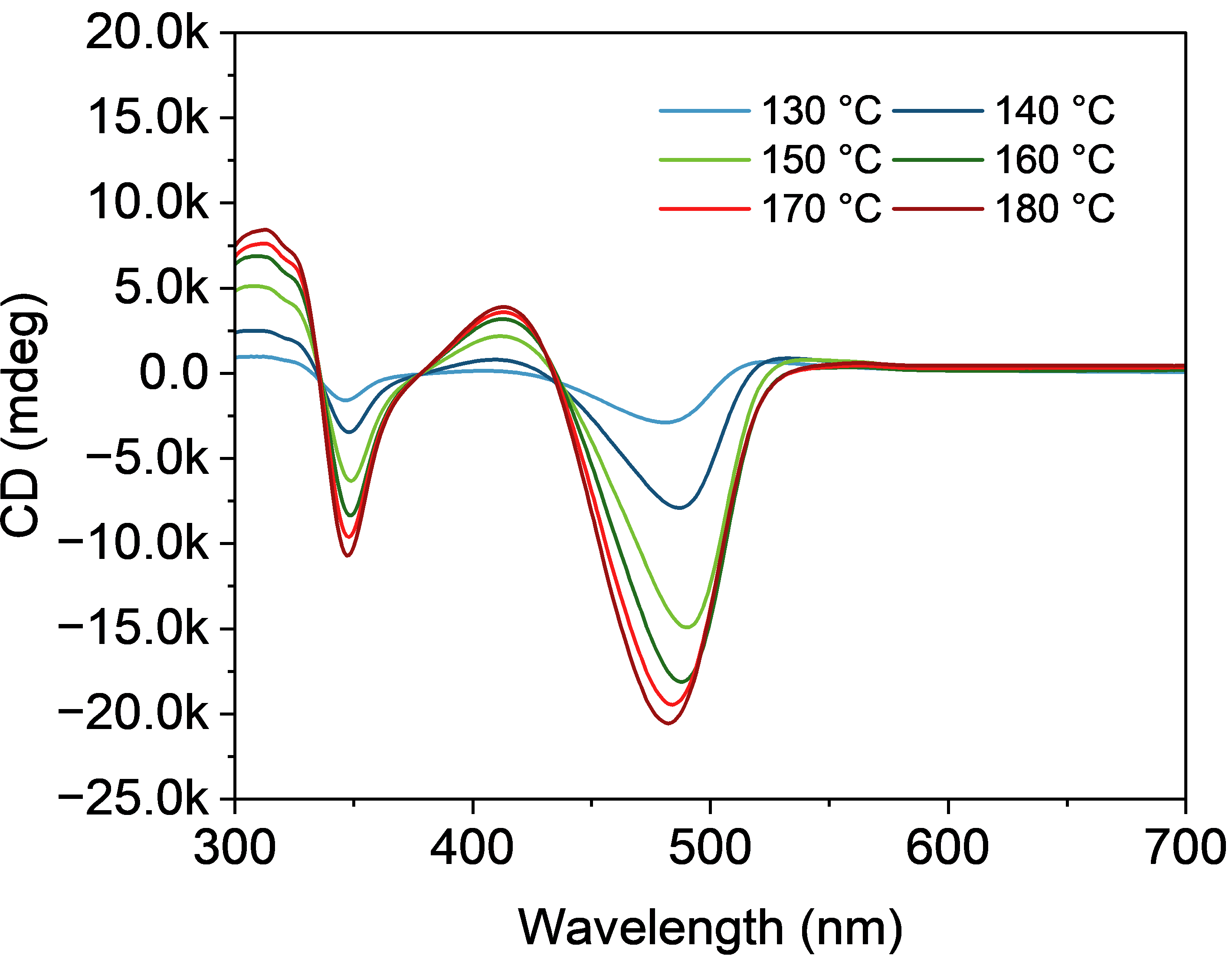


**Fig. S5** Temperature dependent CD spectra of a [*M*,*M*]-PD8H-6R:F8BT blend indicating the formation of chiral F8BT environments for the double helicene emitter.


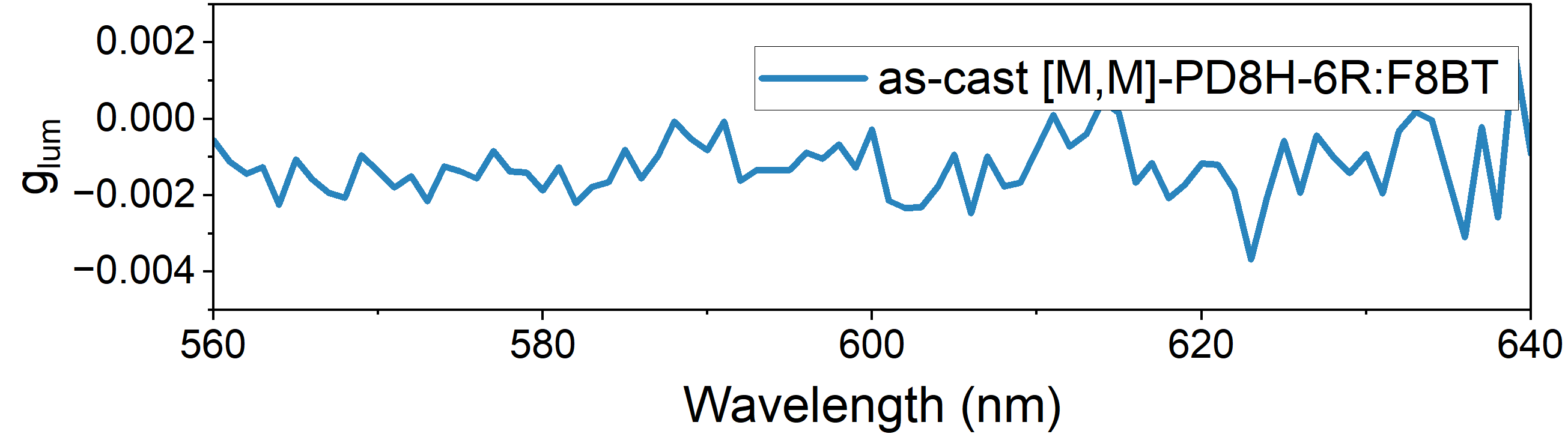


**Fig. S6** CPL and g_lum_ profile of as-cast [*M*,*M*]-PD8H-6R:F8BT blend film


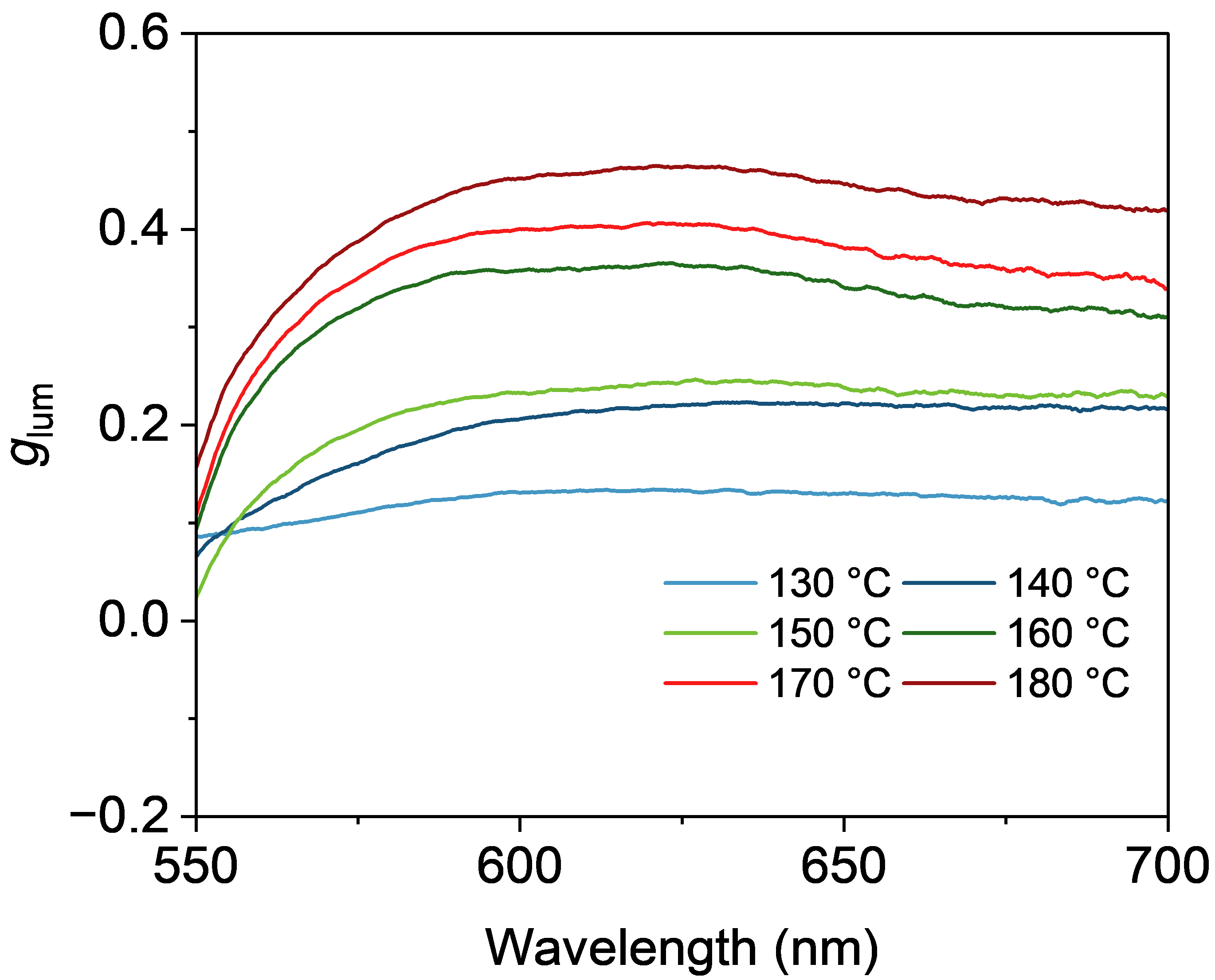


**Fig. S7** Evolution of the *g*_lum_ profile of [*M*,*M*]-PD8H-6R:F8BT upon thermal annealing.


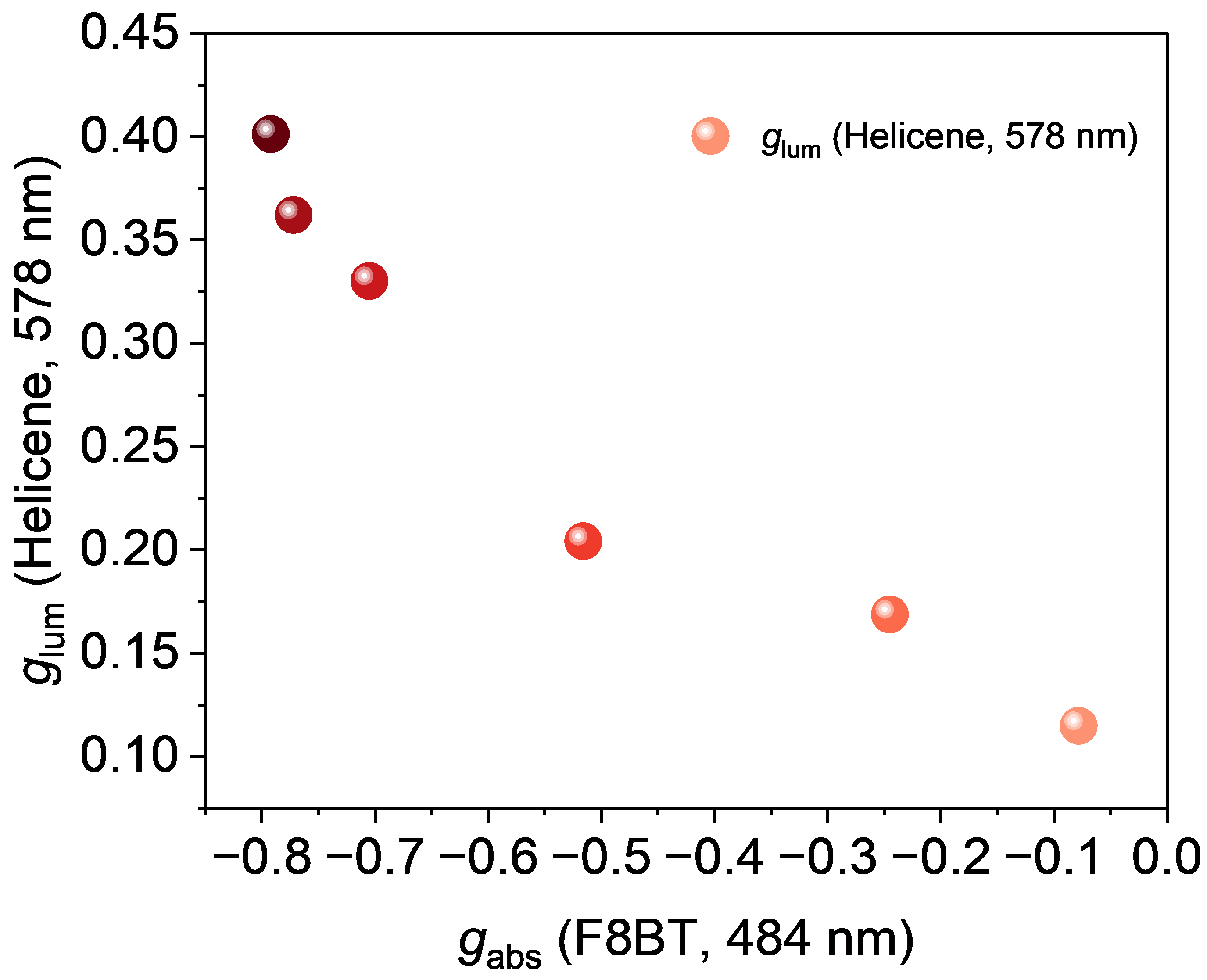


**Fig. S8** Evolution of *g*_lum_ as a function of g_abs_ in F8BT across the annealing temperature range of 130°C to 180 °C.

**Table S4** PLQY of F8BT and PD8H-6R films used in this work

| Samples | F8BT | F8BT, Annealed | [*M*,*M*]-PD8H-6R | [*M*,*M*]-PD8H-6R, Annealed | [*M*,*M*]-PD8H-6R in F8BT | [*M*,*M*]-PD8H-6R in F8BT, annealed |
| --- | --- | --- | --- | --- | --- | --- |
| PLQY (%) | 54±2 | 62±3 | 17±2 | 13±2 | 40±2 | 47±3 |


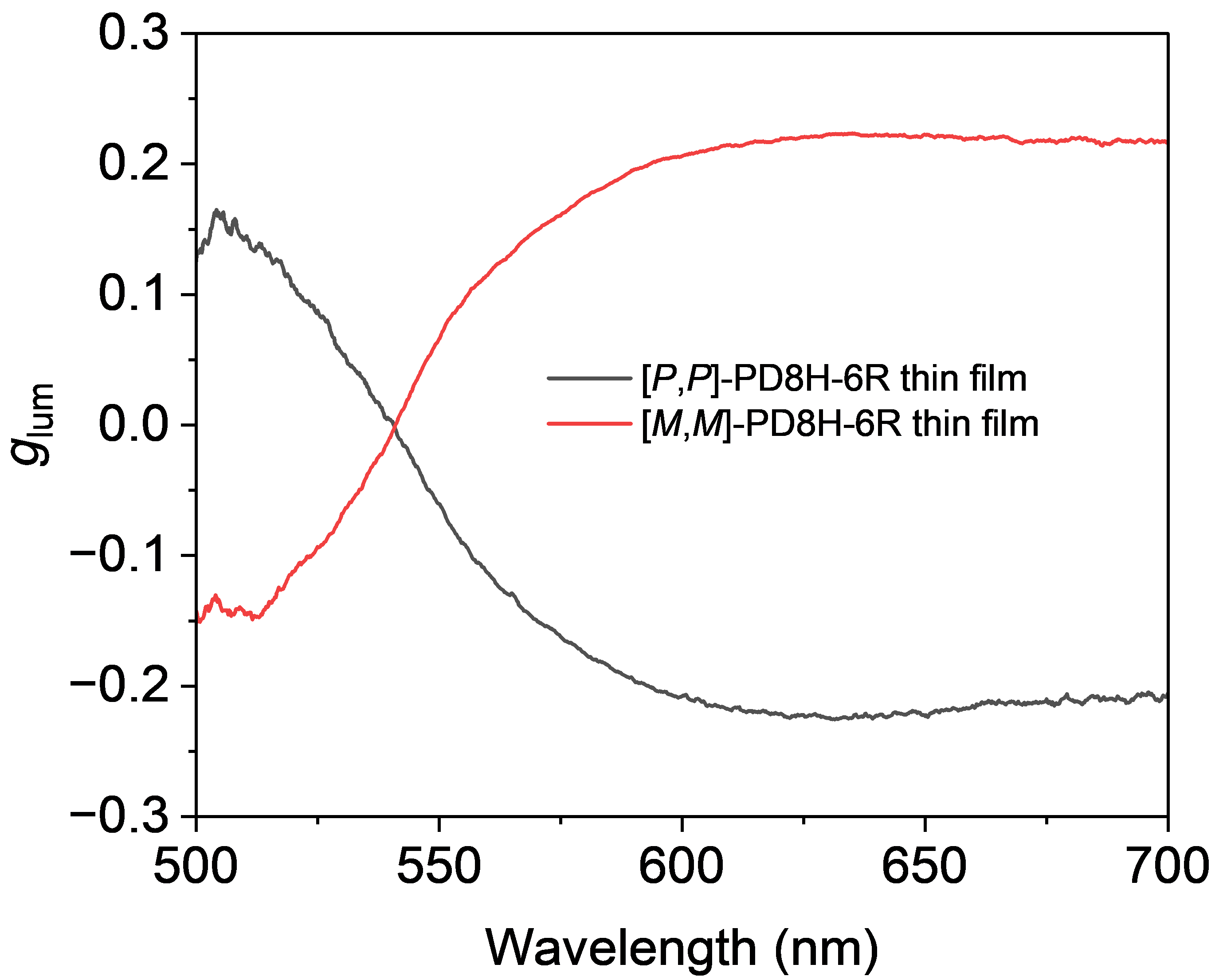


**Fig. S9** [*M*,*M*]- and [*P*,*P*]-PD8H-6R:F8BT blend CPL g-factor profiles. These samples were annealed at 140 °C.


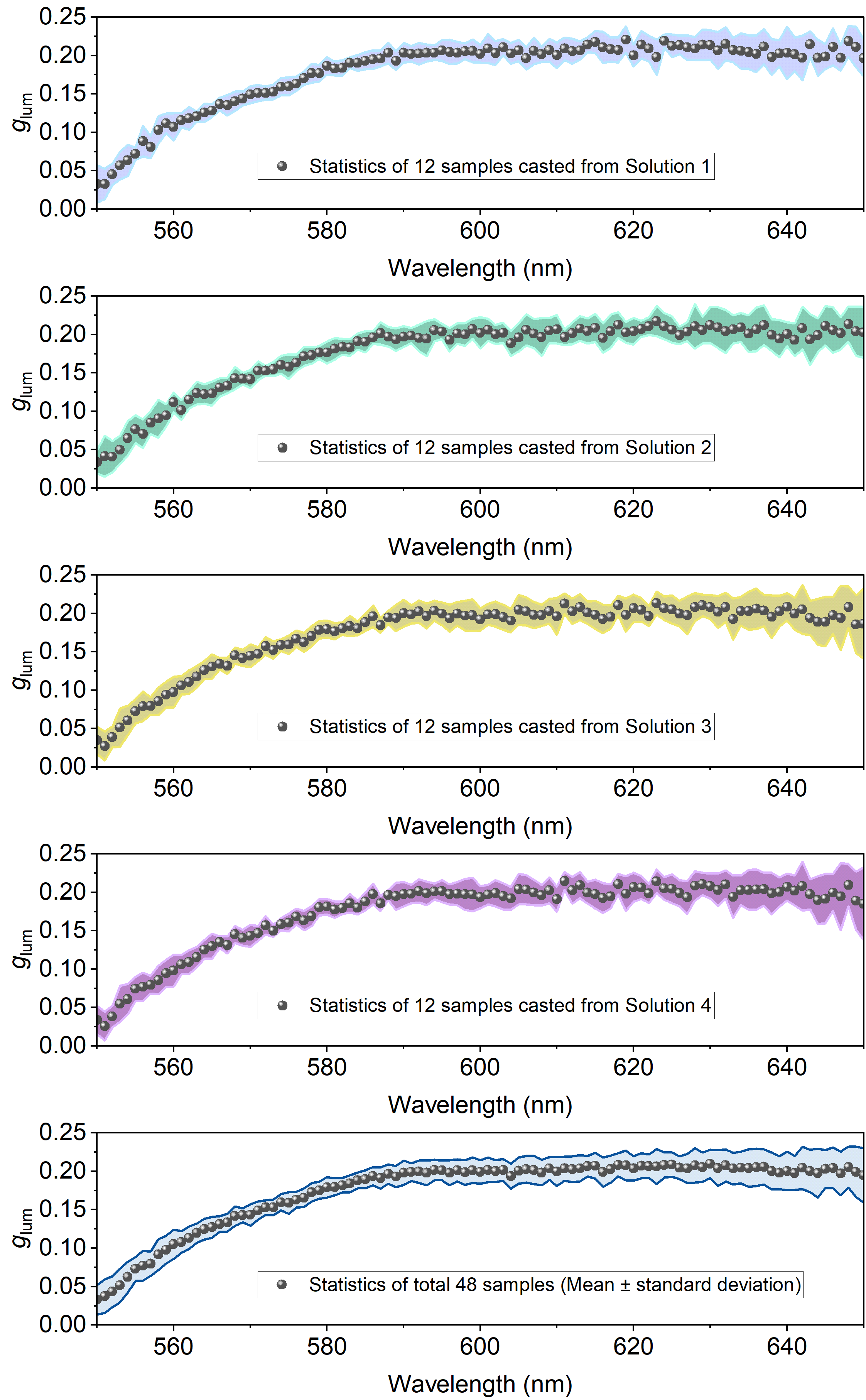


**Fig. S10** Reproducibility test of F8BT:PD8H blend CPL scans. All plots were displayed as Mean ± standard deviation. Error bars are displayed as error bands in graphs as shaded area.

**Table S5** Statistic Report of F8BT:PD8H blend CPL measurements

| Sample | *g*_lum_^a^ @580 nm (mean ± std) | *g*_lum_ @623 nm (mean ± std) |
| --- | --- | --- |
| 12 Samples from Solution 1 | 0.187 ± 0.011 | 0.198 ± 0.023 |
| 12 Samples from Solution 2 | 0.177 ± 0.014 | 0.217 ± 0.020 |
| 12 Samples from Solution 3 | 0.180 ± 0.011 | 0.213 ± 0.015 |
| 12 Samples from Solution 4 | 0.181 ± 0.010 | 0.214 ± 0.016 |
| Total 48 Samples | 0.179 ± 0.013 | 0.206 ± 0.023 |

^a^ The detection limit of *g*_lum_ for the equipment is 0.00001, our signal is far stronger than this value, two significant figures will be enough as reported in main manuscript.

**
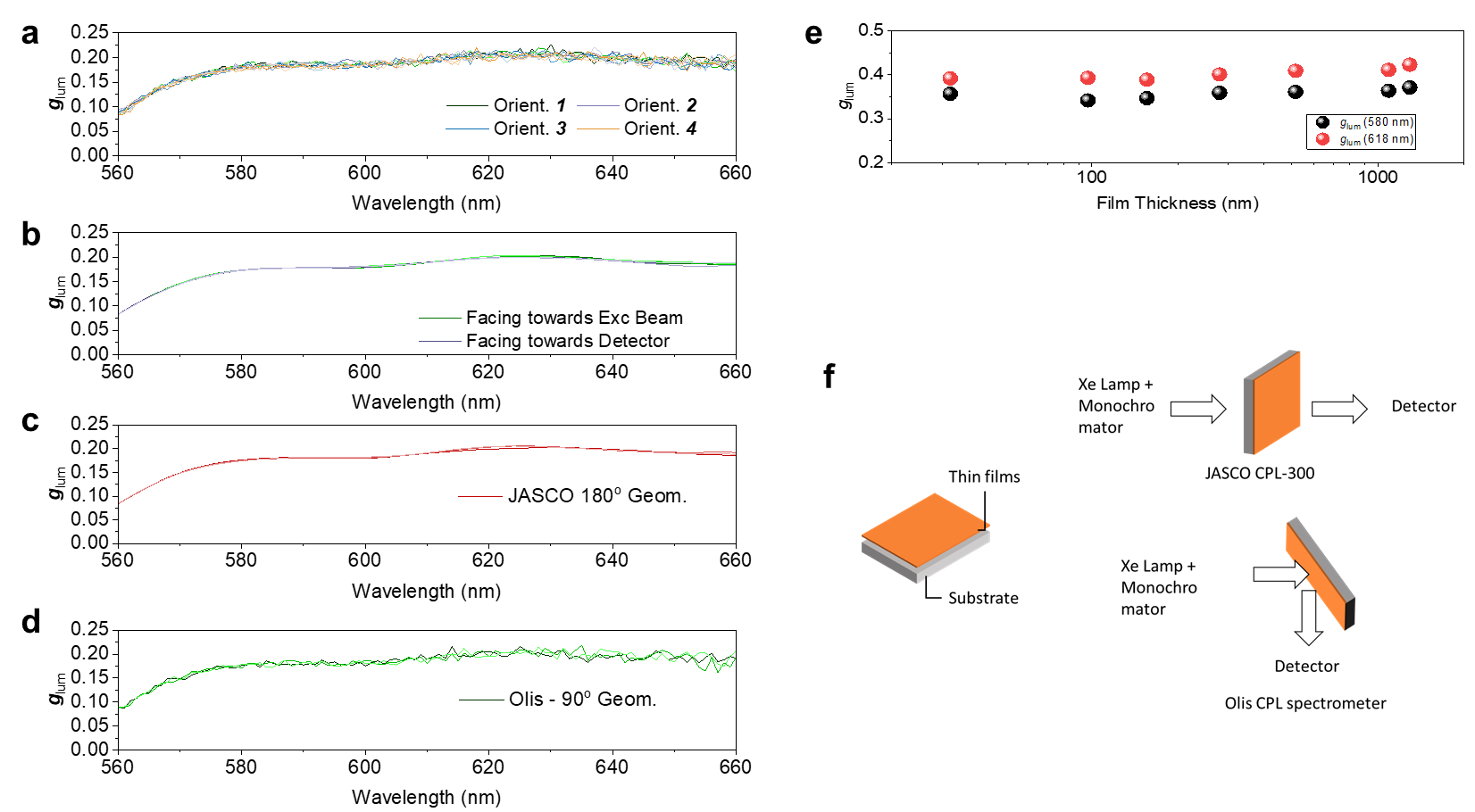
**

**Fig. S11** CPL performance of [*M*,*M*]-PD8H-6R:F8BT blend annealed at 140 °C. a. Orientation dependent CPL measurements performed using an Olis CPL spectrometer. Thin films were rotated to measure in different orientations. b. CPL performance check by flipping the sample. Data recorded using a JASCO CPL-300 spectrometer. c. and d. compared CPL profiles that were recorded using two different commercially available spectrometer, indicating the CPL performance is independent of measurement geometry. e. CPL performance of blend films with wide thickness range, measured using Olis 90° geometry. f. Demonstration of measurement geometry for JASCO and Olis CPL spectrometer. JASCO CPL-300 uses a 180° geometry while Olis CPL spectrometer uses a 90° geometry.


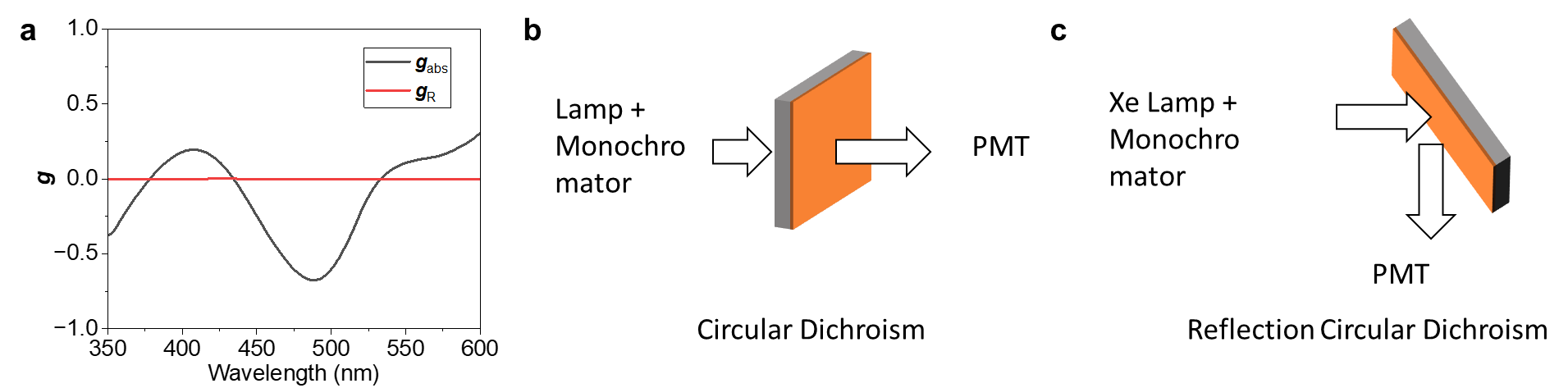


**Fig. S12** Circular dichroism and Reflection Circular Dichroism measurements: a. Dissymmetry factor extracted from circular dichroism (*g*_abs_) and reflection circular dichroism (*g*_R_); b. and c. represent the measurement geometry of circular dichroism and reflection circular dichroism.


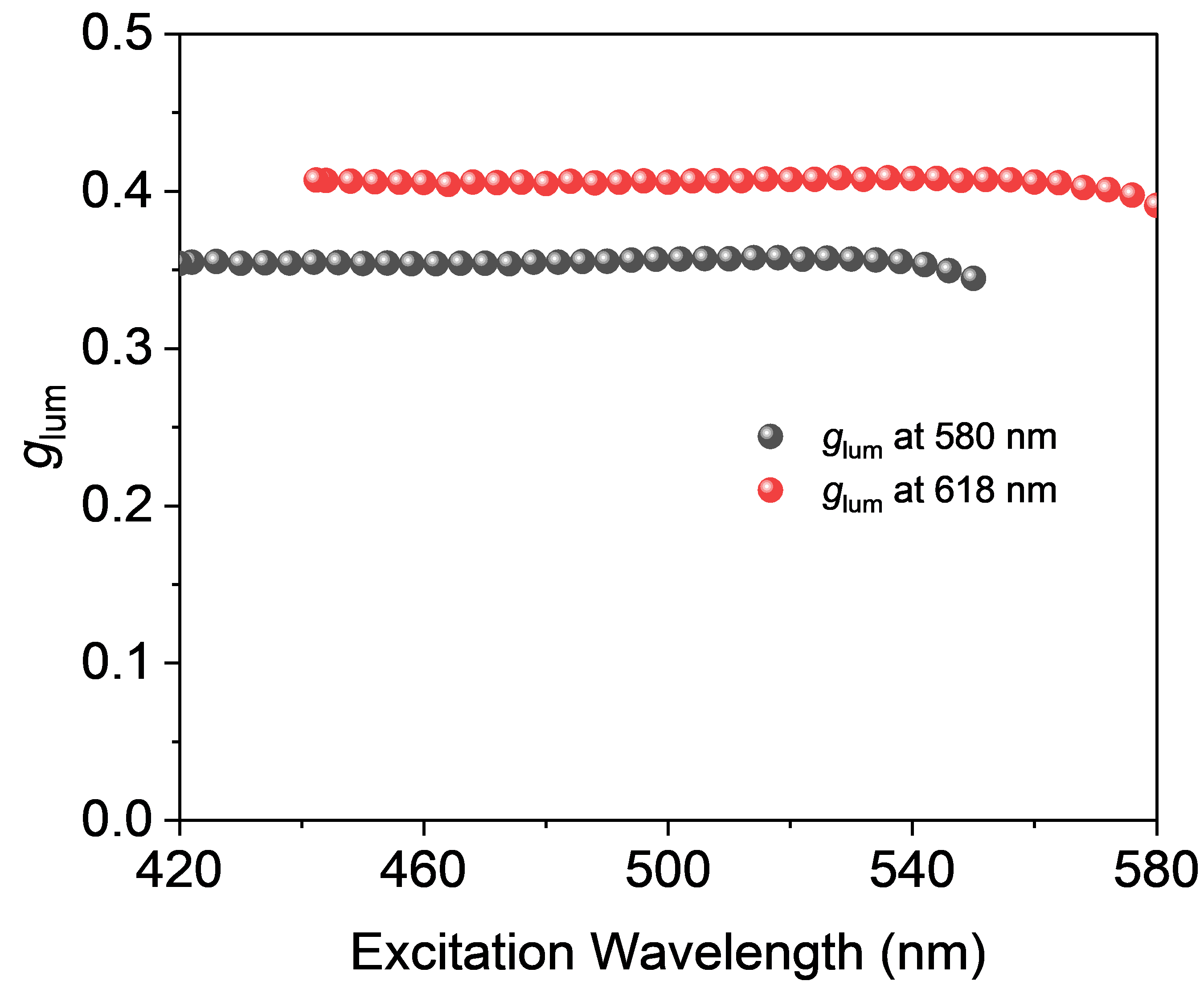


**Fig. S13** CPLE spectra of [*M*,*M*]-PD8H-6R:F8BT. The CPL signals were probed for the first and second vibronic emission peaks.


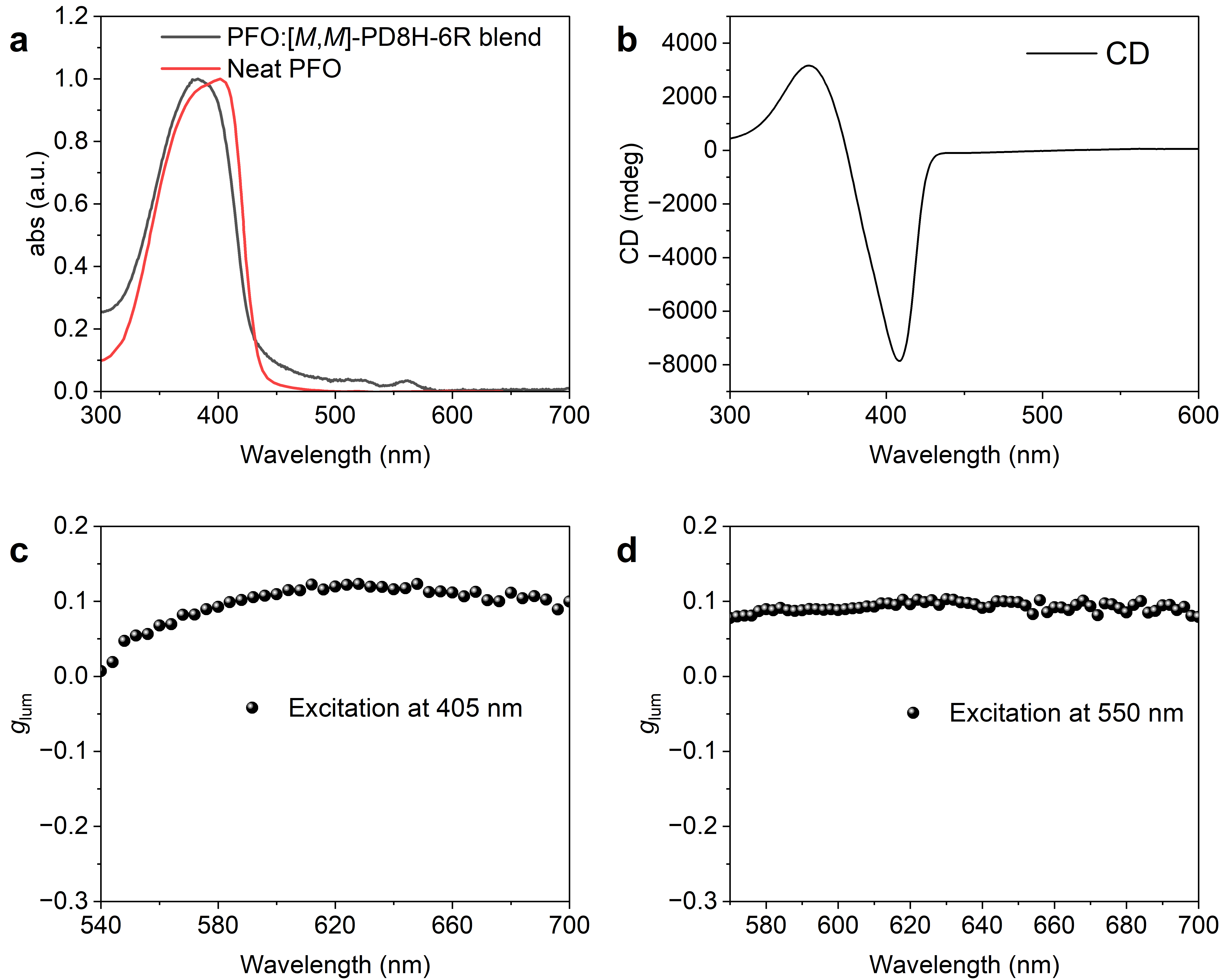


**Fig. S14** CPL amplification of [*M*,*M*]-PD8H-6R using another polyfluorene, PFO. a. Absorption profile of PFO and the PFO:[*M*,*M*]-PD8H-6R blend; b. CD profile of the PFO:[*M*,*M*]-PD8H-6R blend; c. *g*_lum_ profile of the PFO:[*M*,*M*]-PD8H-6R blend CPL emission at an excitation wavelength of 405 nm; *g*_lum_ profile of the PFO:[*M*,*M*]-PD8H-6R blend CPL emission at an excitation wavelength of 550 nm


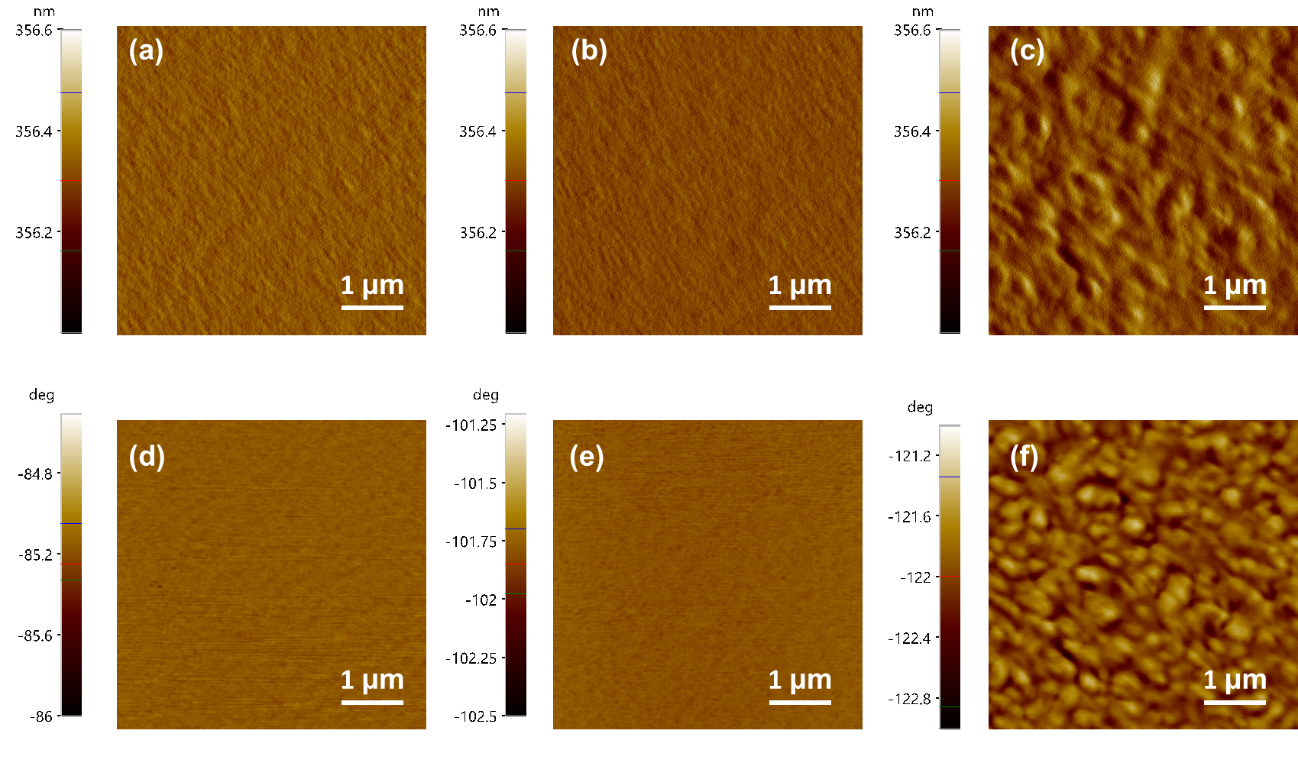


**Fig. S15** AFM images of F8BT and blend films. (a)(b)(c) Amplitude and (d)(e)(f) Phase imaging of (a)(b) Neat F8BT, (c)(d) as-cast F8BT:PD8H blend film, and (e)(f) annealed F8BT:PD8H blend film


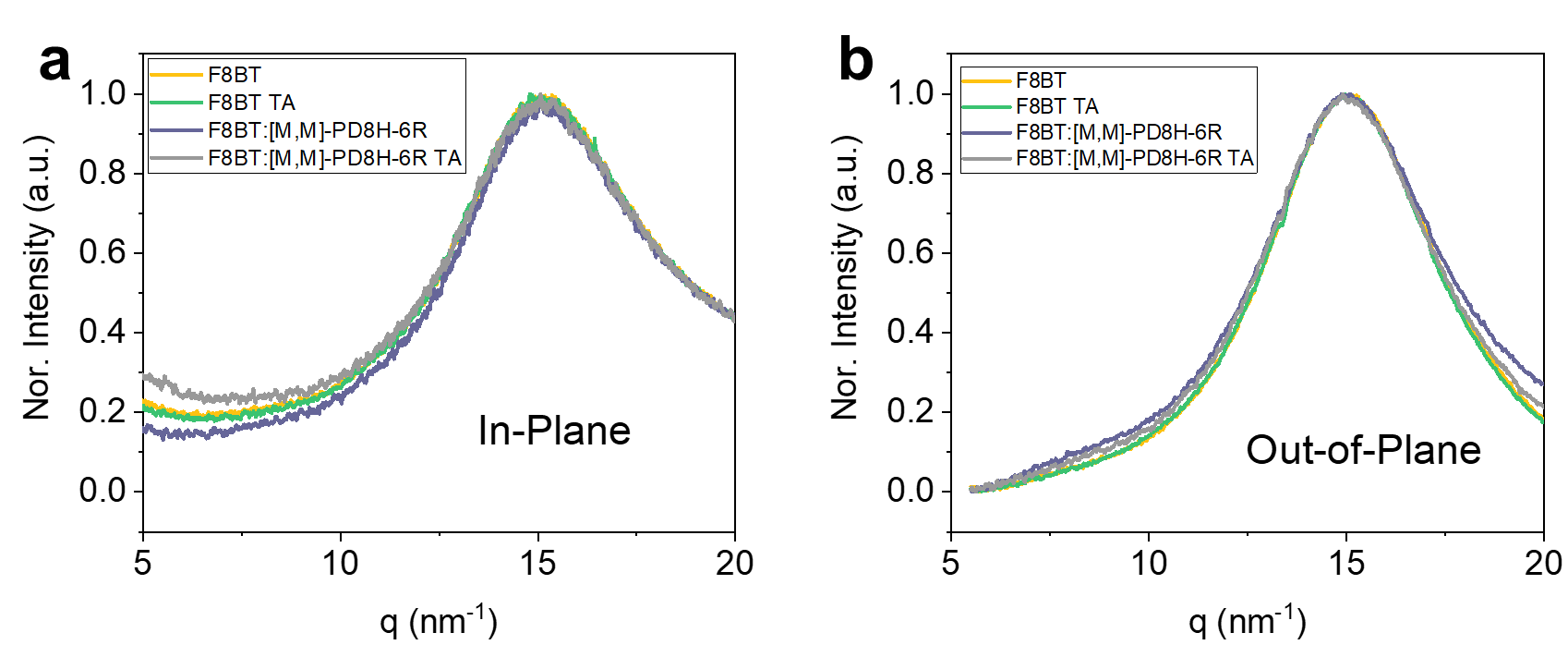


**Fig. S16** 2D-GIWAXS profile of F8BT and F8BT:[*M*,*M*]-PD8H-6R blend films before and after thermal annealing (TA).


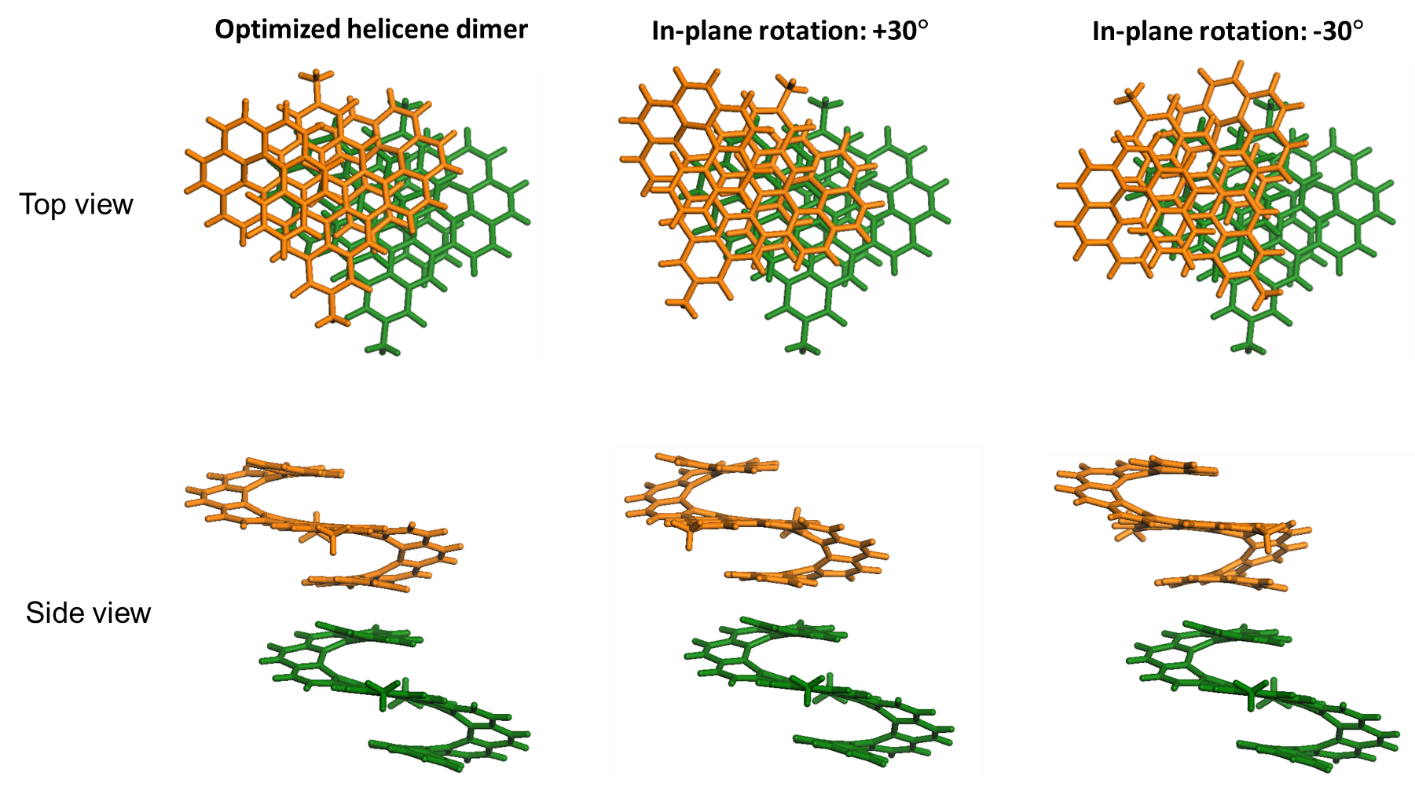


**Fig. S****17** Illustration of the optimized [*M*,*M*]-PD8H-6R helicene dimer and rotated forms. The helicene dimer including full side chains was first optimized at the GFN2-xTB level^5^ with the xTB software^6^; then, the side chains were replaced with H atoms and one of the molecules was rotated by +30° and –30°.

**Table S6** Calculated excited-state properties of [*M*,*M*]-PD8H-6R dimers represented in Fig. S17, compared to those of a single [*M*,*M*]-PD8H-6R molecule.


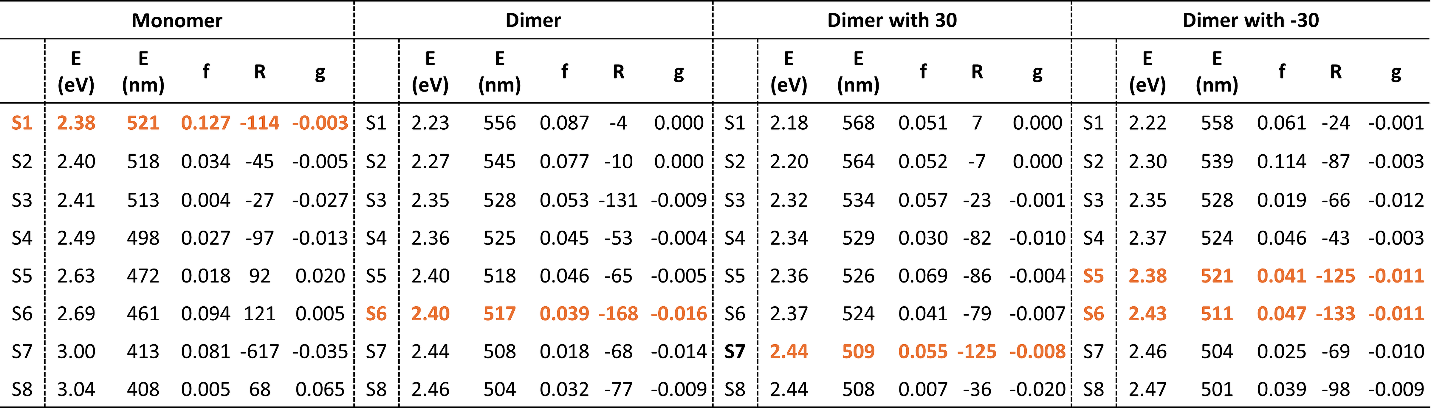


Self-aggregations of [*M*,*M*]-PD8H-6R molecules do not change the sign of *R* and have negligible effects on the associated *g*-factor*.*


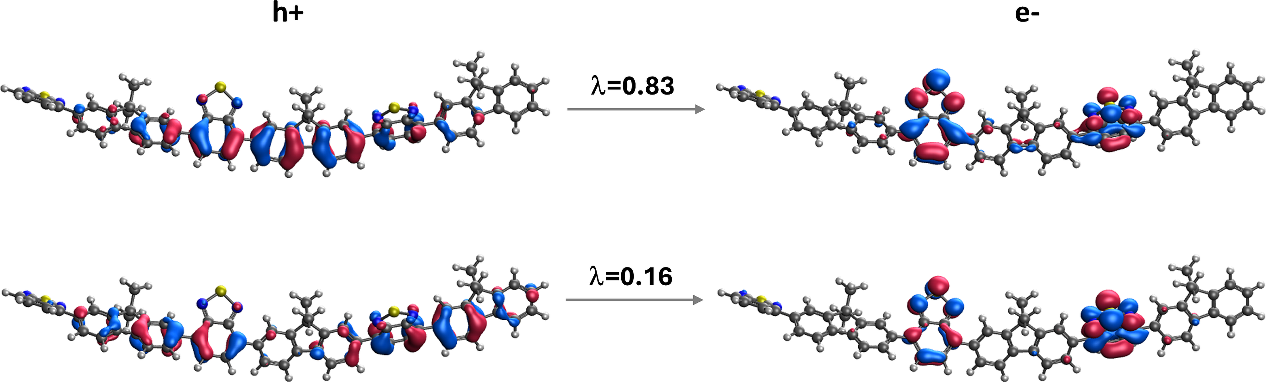


**Fig. S18** Natural Transition Orbitals (NTOs) in the S_1_ state of a single F8BT oligomer consisting of 3 repeat units, as calculated at the LC-ωHPBE/6-31G** level and considering the optimized ground-state geometry. S_1_: E = 2.64 eV (469 nm), f= 1.558, R=130 × 10^−40^ esu cm erg G^-1^, *g* = 0.000.


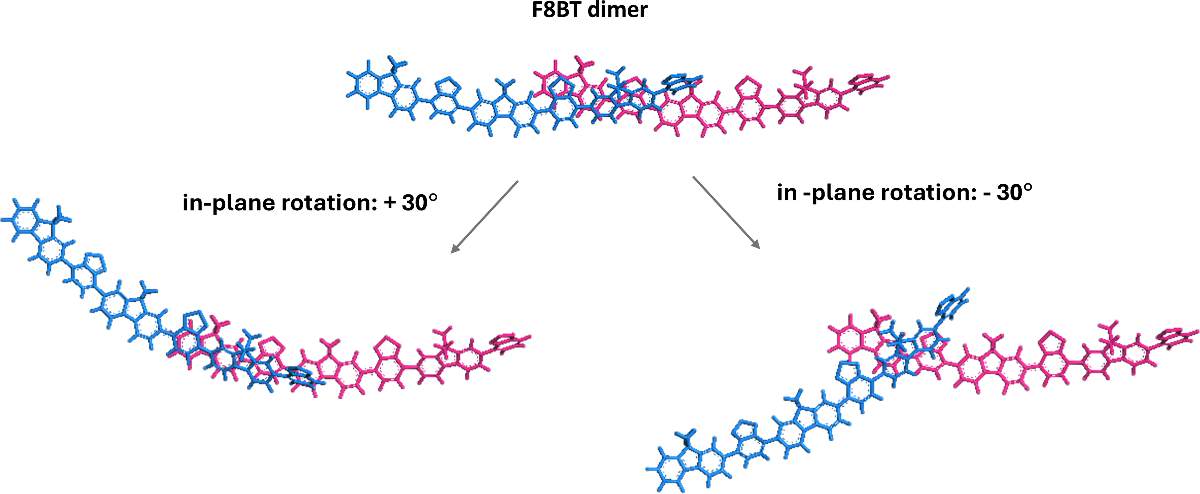


**Fig. S****19** Illustration of the F8BT dimer and its rotated forms. Two optimized F8BT monomers form the dimer by positioning them in a 0° in-plane orientation. One of the molecules was then rotated by +30° and –30°.

**Table S7** Calculated excited-state properties of F8BT dimers represented in Fig. S19.

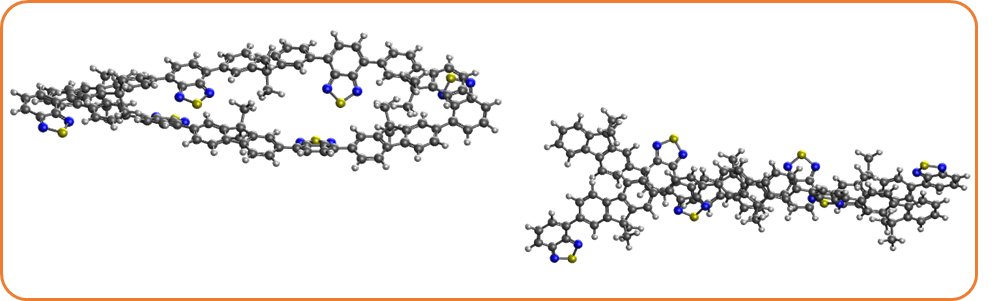


**Fig. S****20** Optimized F8BT dimer at the GFN2-xTB level^5^ with the xTB software^6^; full side chains are considered for the geometry optimization and then replaced with hydrogen atoms for the TD-DFT calculations.

**Table S8** Calculated chiral properties of the F8BT dimer represented in Fig. S20.

|  | E  (eV) | E  (nm) | *f* | \|*μ*\|  (10^−20^ esu cm) | \|*m*\|  (10^−20^ erg G^−1^) | Cos(*θ*^b^) | *R*  (10^−40^ erg  esu cm G^−1^) | *g* |
| --- | --- | --- | --- | --- | --- | --- | --- | --- |
| S_0_ → S_1_ | 2.37 | 524 | 0.046 | 225 | 4.87 | 0.57 | 623 | 0.049 |
| S_0_ → S_2_ | 2.42 | 512 | 1.380 | 1226 | 1.70 | -0.03 | -69 | 0.000 |
| S_0_ → S_3_ | 2.50 | 495 | 0.945 | 999 | 2.70 | -0.19 | -519 | -0.002 |

**Table S****9** Calculated electronic transitions and chiral characteristics of the F8BT dimer / helicene complex with 0° between ***µ***(helicene) and ***µ***(F8BT), as shown in Figure 4.

|  | E  (eV) | E  (nm) | *f* | \|***μ***\|  (10^−20^ esu cm) | \|***m***\|  (10^−20^ erg G^−1^) | Cos(*θ*^b^) | *R*  (10^−40^ erg  esu cm G^−1^) | *g* |
| --- | --- | --- | --- | --- | --- | --- | --- | --- |
| S_0_ → S_4_ | 2.40 | 516 | 0.028 | 176 | 0.45 | -0.40 | -31 | -0.004 |
| S_0_ → S_5_ | 2.41 | 515 | 1.257 | 1173 | 1.67 | -0.05 | -105 | 0.000 |
| S_0_ → S_6_ | 2.41 | 514 | 0.023 | 159 | 0.91 | -0.28 | -40 | -0.006 |


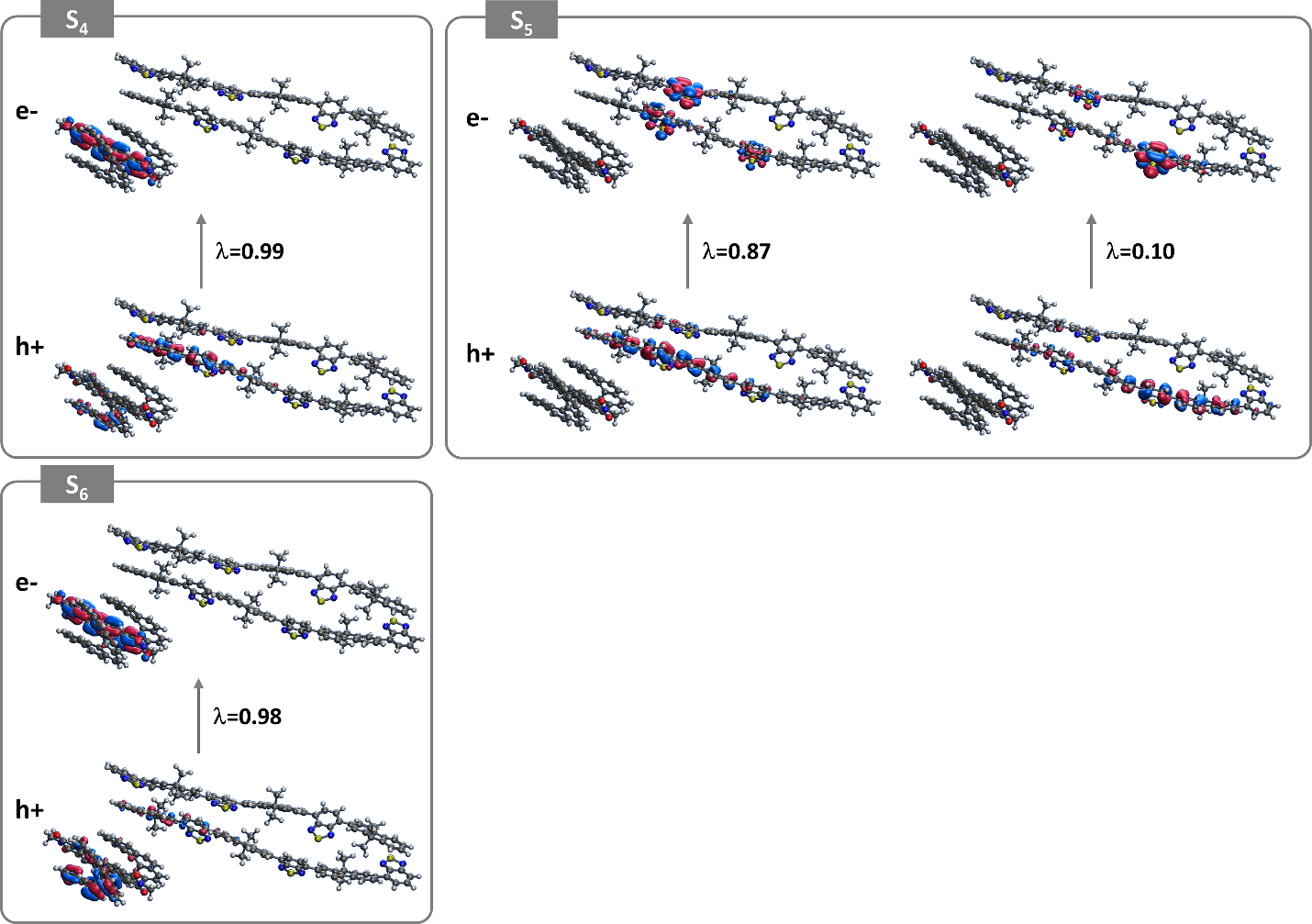


**Fig. S21** Natural Transition Orbitals (NTOs) in the S_4_, S_5_, and S_6_ states of the complex with 0° between ***µ***(helicene) and ***µ***(F8BT), as shown in Figure 4 (see also Table S9).


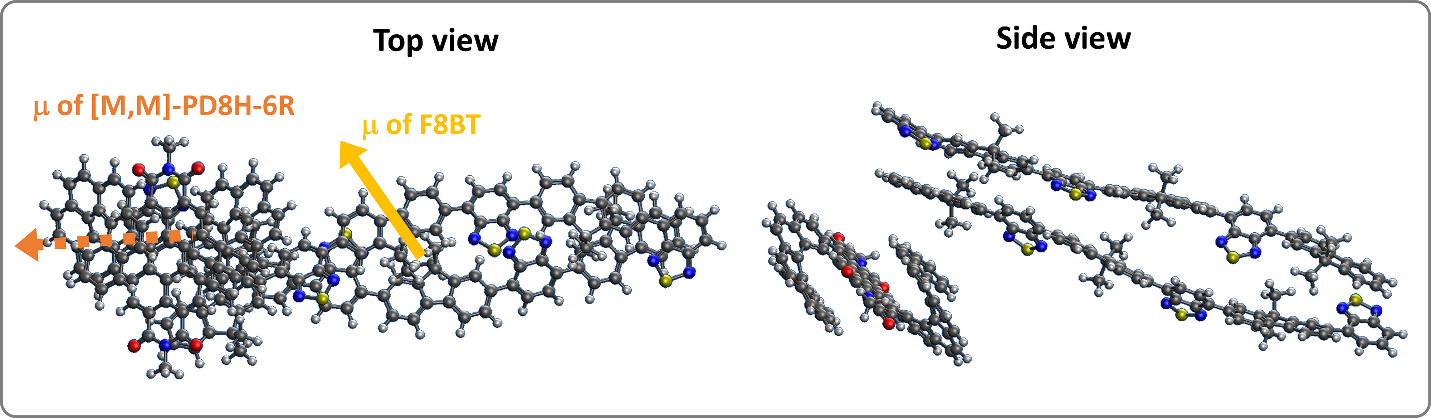


**Fig. S****22** Complex consisting of an F8BT dimer and an [*M*,*M*]-PD8H-6R molecule with a –60° angle between ***µ***(helicene) and ***µ***(F8BT).

**Table S****10** Calculated electronic transitions and chiral characteristics of the F8BT dimer / helicene complex with a –60° angle between ***µ***(helicene) and ***µ***(F8BT), as shown in Fig. S22.

|  | E  (eV) | E  (nm) | *f* | \|***μ***\|  (10^−20^ esu cm) | \|***m***\|  (10^−20^ erg G^−1^) | Cos(*θ*^b^) | *R*  (10^−40^ erg  esu cm G^−1^) | *g* |
| --- | --- | --- | --- | --- | --- | --- | --- | --- |
| S_0_ → S_1_ | 2.36 | 524.77 | 0.01 | 88 | 3.51 | 0.86 | 265 | 0.136 |
| S_0_ → S_2_ | 2.37 | 523.94 | 0.09 | 324 | 2.87 | 0.32 | 301 | 0.011 |
| S_0_ → S_3_ | 2.39 | 519.80 | 0.09 | 321 | 1.43 | -0.36 | -164 | -0.006 |
| S_0_ → S_4_ | 2.39 | 518.36 | 0.00 | 22 | 0.17 | -0.15 | -1 | -0.005 |
| S_0_ → S_5_ | 2.41 | 513.51 | 1.21 | 1150 | 1.30 | 0.06 | 97 | 0.000 |
| S_0_ → S_6_ | 2.42 | 512.81 | 0.04 | 211 | 1.52 | -0.23 | -75 | -0.007 |

The S_1_ state has a hybrid character with a 32% contribution from electronic transitions on helicene and 62% on F8BT; the S_2_ state is also hybrid with a 66% contribution from electronic transitions on helicene and 27% on F8BT (see Fig. S23).


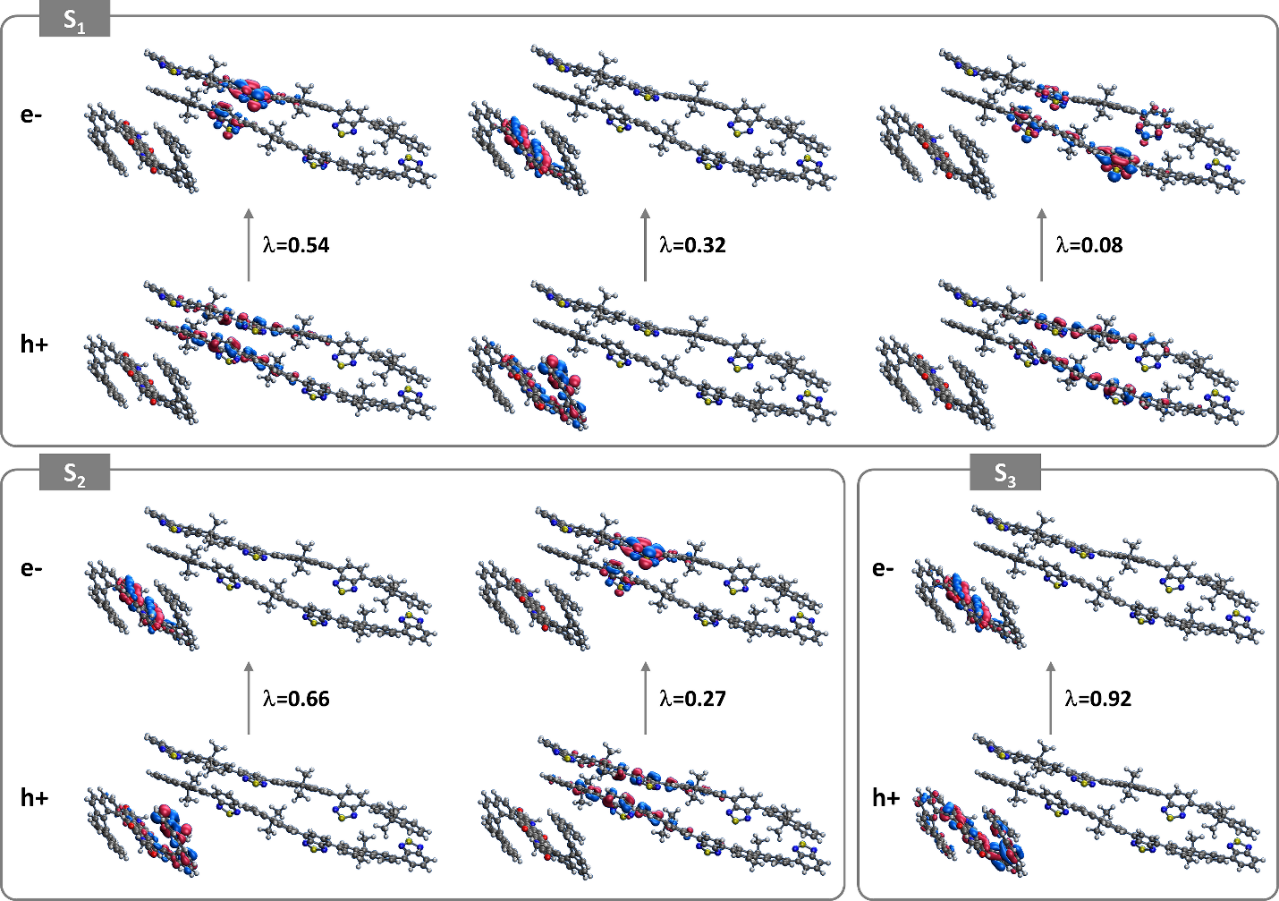


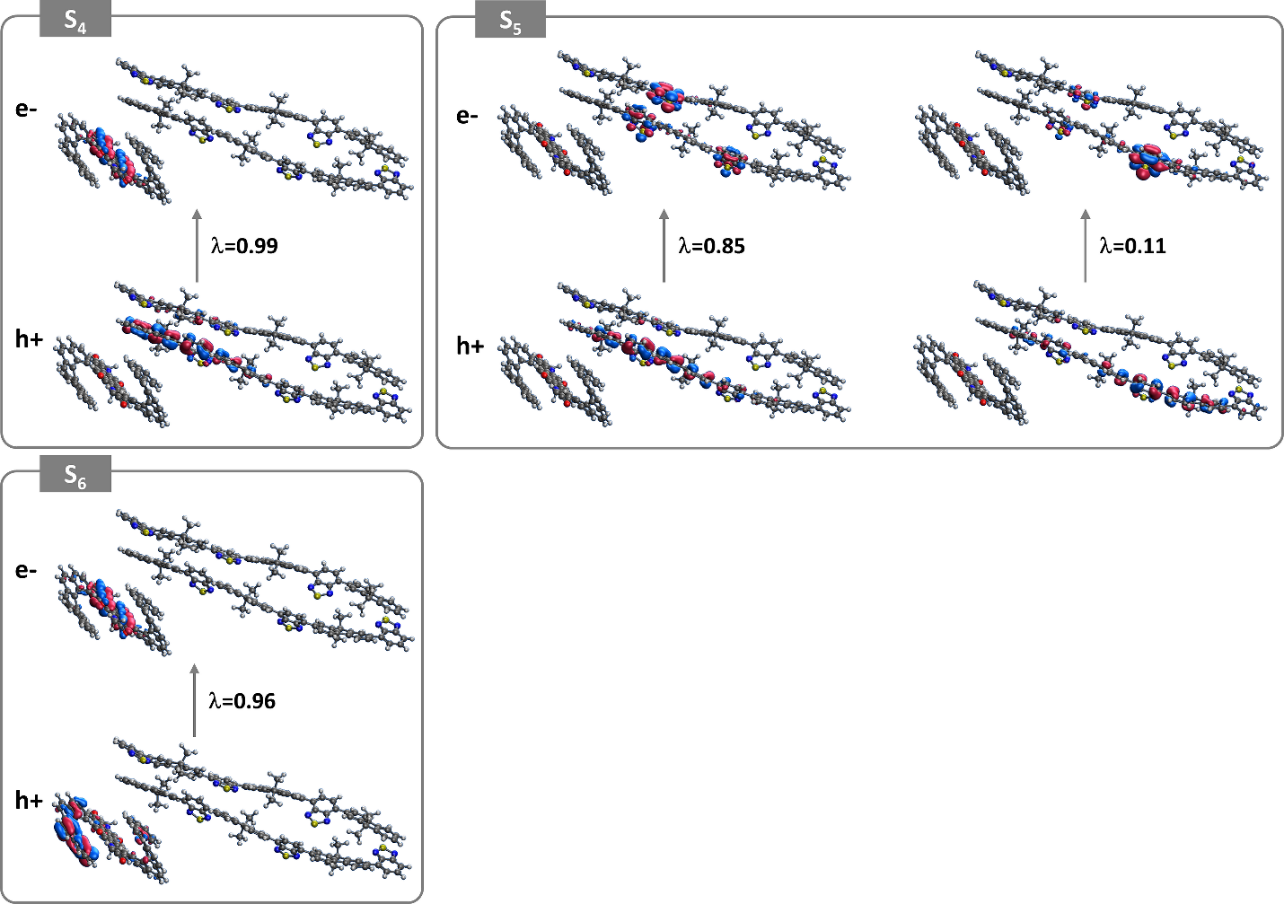


**Fig. S****23** Natural Transition Orbitals (NTOs) in the six lowest singlet states of the complex with a –60° angle between ***µ***(helicene) and ***µ***(F8BT), as shown in Fig. S22 (see also Table S10).


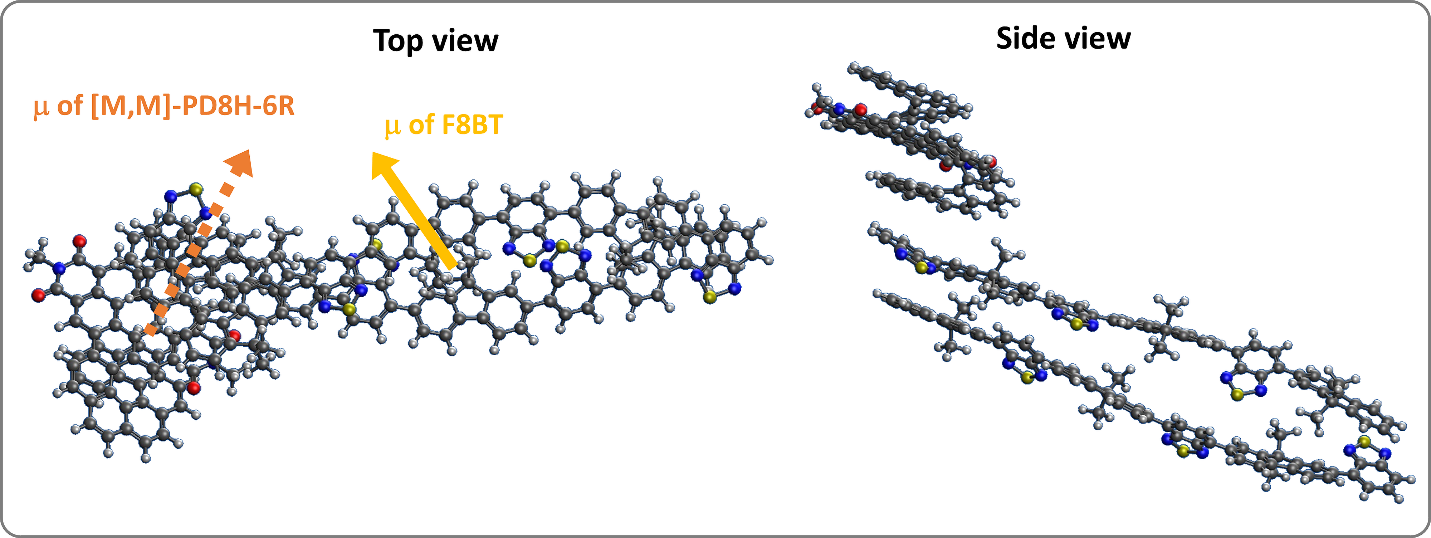


**Fig. S****24** Complex consisting of an F8BT dimer and an [*M*,*M*]-PD8H-6R helicene molecule with a +60° angle between ***µ***(helicene) and ***µ***(F8BT).

**Table S****11** Calculated electronic transitions and chiral characteristics of the F8BT dimer / helicene complex with a +60° angle between ***µ***(helicene) and ***µ***(F8BT), as shown in Fig. S24.

|  | E  (eV) | E  (nm) | *f* | \|***μ***\|  (10^−20^ esu cm) | \|***m***\|  (10^−20^ erg G^−1^) | Cos(*θ*^b^) | *R*  (10^−40^ erg  esu cm G^−1^) | *g* |
| --- | --- | --- | --- | --- | --- | --- | --- | --- |
| S_0_ → S_1_ | 2.36 | 524.93 | 0.07 | 288 | 3.14 | 0.58 | 523 | 0.025 |
| S_0_ → S_2_ | 2.37 | 522.27 | 0.05 | 247 | 2.69 | 0.23 | 155 | 0.010 |
| S_0_ → S_3_ | 2.38 | 520.34 | 0.17 | 429 | 2.76 | 0.04 | 47 | 0.001 |
| S_0_ → S_4_ | 2.40 | 516.75 | 0.04 | 212 | 1.97 | -0.23 | -96 | -0.009 |
| S_0_ → S_5_ | 2.42 | 512.68 | 1.37 | 1224 | 3.64 | -0.07 | -305 | -0.001 |
| S_0_ → S_6_ | 2.42 | 511.70 | 0.01 | 102 | 0.94 | 0.87 | 83 | 0.032 |

The S_1_ state hybridization consists of an 8% contribution from helicene and 86% from F8BT. The S_2_ state is an electronic transition from F8BT to helicene. The S_3_ and S_6_ states are localized on the helicene but have a sign inverted g factor (see also Fig. S25).


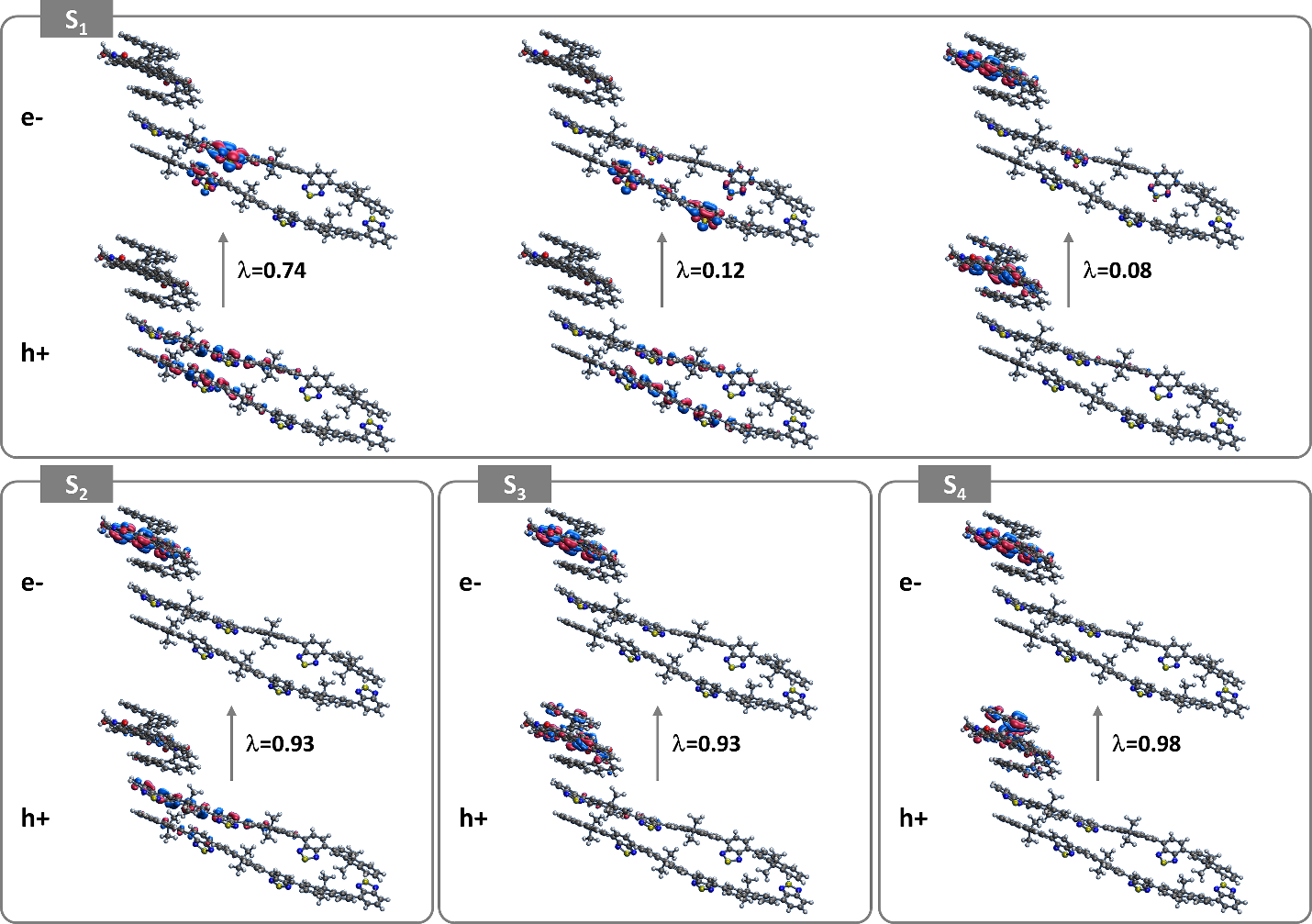


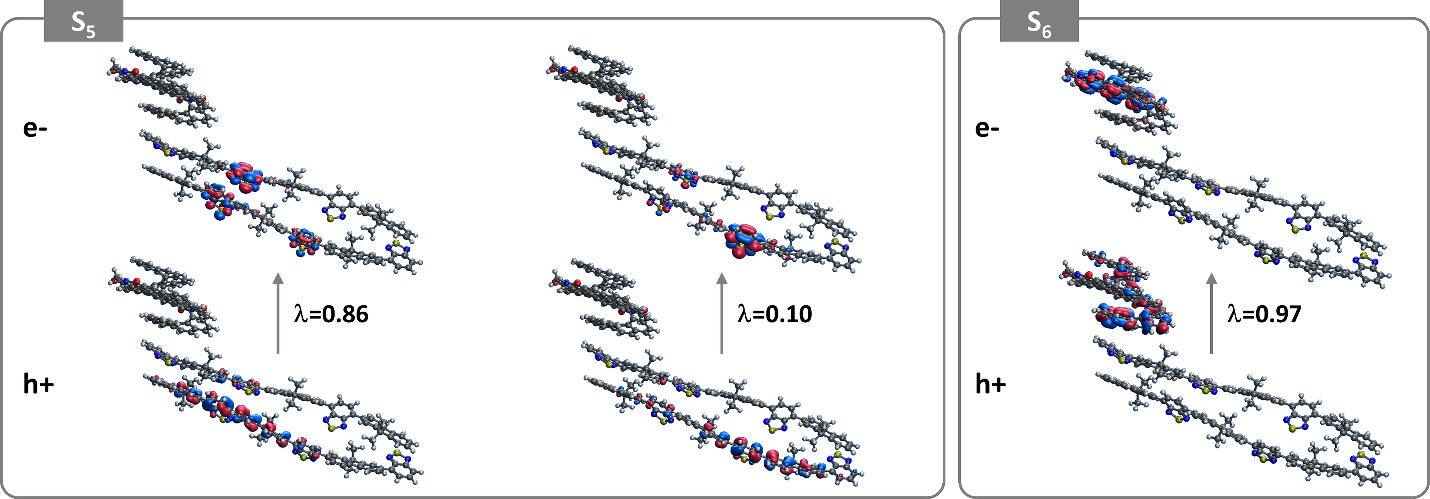


**Fig. S****25** Natural Transition Orbitals (NTOs) in the six lowest singlet states of the complex with a +60° angle between ***µ***(helicene) and ***µ***(F8BT), as shown in Figure S21 (see also Table S11).

**Further discussion:**

Transient absorption or transient PL would be useful to evaluate state dynamics; however, neither of these techniques can be taken as a direct probe of hybridized states, as changes in excited-state dynamics can also be caused by other structural or excitonic interactions. Nevertheless, we show below transient absorption data for both annealed and non-annealed blend films as supporting evidence for excited-state hybridization. We excited the film at λ = 565 nm to ensure that only the helicene is excited in the blend and probed the helicene excited states. By performing a line cut at 660 nm for the 2D spectra, we observed a clear difference in dynamics for the helicene excited states. We used a bi-exponential decay model to fit the spectra and found that after annealing the excited-state lifetime dropped significantly. This change in state dynamics is further evidence of excited-state hybridization.


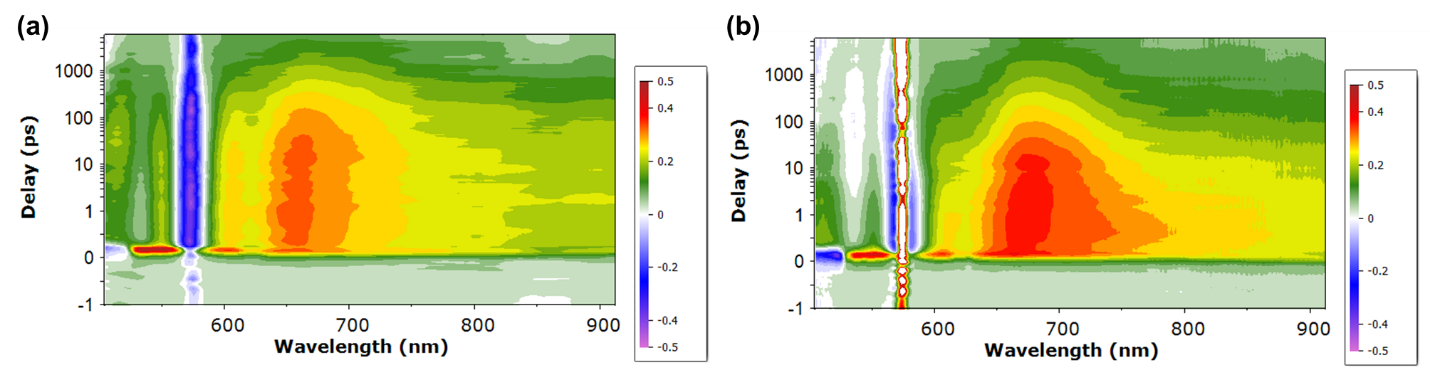


**Fig. S26** Transient absorption spectra of (a) non-annealed and (b) annealed F8BT: [*M*,*M*]-PD8H-6R blend films excited at 565 nm.


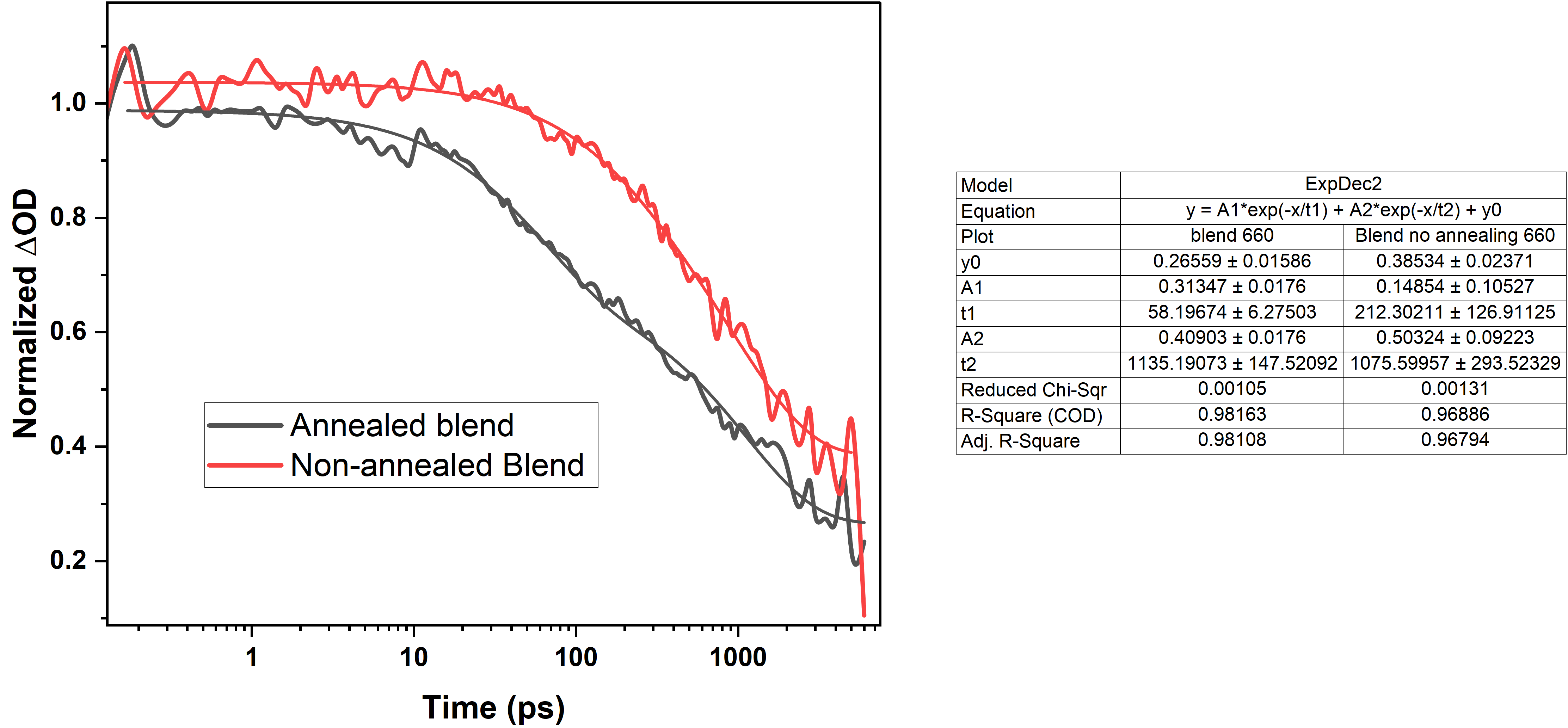


**Fig. S27** Dynamics at 660 nm, extracted from Fig.S26.

(1) Han, J.; Duan, P.; Li, X.; Liu, M. Amplification of Circularly Polarized Luminescence through Triplet–Triplet Annihilation-Based Photon Upconversion. *J. Am. Chem. Soc.* **2017**, *139* (29), 9783–9786. https://doi.org/10.1021/jacs.7b04611.

(2) Han, D.; Yang, X.; Han, J.; Zhou, J.; Jiao, T.; Duan, P. Sequentially Amplified Circularly Polarized Ultraviolet Luminescence for Enantioselective Photopolymerization. *Nat. Commun.* **2020**, *11* (1). https://doi.org/10.1038/s41467-020-19479-1.

(3) Wade, J.; Brandt, J. R.; Reger, D.; Zinna, F.; Amsharov, K. Y.; Jux, N.; Andrews, D. L.; Fuchter, M. J. 500-Fold Amplification of Small Molecule Circularly Polarised Luminescence through Circularly Polarised FRET. *Angew. Chemie Int. Ed.* **2021**, *60* (1), 222–227. https://doi.org/10.1002/ANIE.202011745.

(4) Liu, B.; Böckmann, M.; Jiang, W.; Doltsinis, N. L.; Wang, Z. Perylene Diimide-Embedded Double [8]Helicenes. *J. Am. Chem. Soc.* **2020**, *142* (15), 7092–7099. https://doi.org/10.1021/JACS.0C00954.

(5) Bannwarth, C.; Ehlert, S.; Grimme, S. GFN2-XTB—An Accurate and Broadly Parametrized Self-Consistent Tight-Binding Quantum Chemical Method with Multipole Electrostatics and Density-Dependent Dispersion Contributions. *J. Chem. Theory Comput.* **2019**, *15* (3), 1652–1671. https://doi.org/10.1021/acs.jctc.8b01176.

(6) Bannwarth, C.; Caldeweyher, E.; Ehlert, S.; Hansen, A.; Pracht, P.; Seibert, J.; Spicher, S.; Grimme, S. Extended Tight‐binding Quantum Chemistry Methods. *WIREs Comput. Mol. Sci.* **2021**, *11* (2), 1–49. https://doi.org/10.1002/wcms.1493.
